# Rice Bran and Probiotics Alter the Porcine Large Intestine and Serum Metabolomes for Protection against Human Rotavirus Diarrhea

**DOI:** 10.3389/fmicb.2017.00653

**Published:** 2017-04-21

**Authors:** Nora Jean Nealon, Lijuan Yuan, Xingdong Yang, Elizabeth P. Ryan

**Affiliations:** ^1^Nutrition and Toxicology Laboratory, Department of Environmental and Radiological Health Sciences, College of Veterinary Medicine and Biomedical Sciences, Colorado State University, Fort CollinsCO, USA; ^2^Yuan Laboratory, Department of Biomedical Sciences and Pathobiology, Virginia-Maryland Regional College of Veterinary Medicine, Virginia Polytechnic Institute and State University, BlacksburgVA, USA; ^3^Laboratory of Infectious Diseases, Viral Pathogenesis and Evolution Section, National Institute of Allergy and Infectious Diseases, National Institute of Health, BethesdaMD, USA

**Keywords:** probiotic, prebiotic, synbiotic, human rotavirus, diarrhea, metabolomics, rice bran, pigs

## Abstract

Human rotavirus (HRV) is a leading cause of severe childhood diarrhea, and there is limited vaccine efficacy in the developing world. Neonatal gnotobiotic pigs consuming a prophylactic synbiotic combination of probiotics and rice bran (Pro+RB) did not exhibit HRV diarrhea after challenge. Multiple immune, gut barrier protective, and anti-diarrheal mechanisms contributed to the prophylactic efficacy of Pro+RB when compared to probiotics (Pro) alone. In order to understand the molecular signature associated with diarrheal protection by Pro+RB, a global non-targeted metabolomics approach was applied to investigate the large intestinal contents and serum of neonatal gnotobiotic pigs. The ultra-high performance liquid chromatography-tandem mass spectrometry platform revealed significantly different metabolites (293 in LIC and 84 in serum) in the pigs fed Pro+RB compared to Pro, and many of these metabolites were lipids and amino acid/peptides. Lipid metabolites included 2-oleoylglycerol (increased 293.40-fold in LIC of Pro+RB, *p* = 3.04E-10), which can modulate gastric emptying, andhyodeoxycholate (decreased 0.054-fold in the LIC of Pro+RB, *p* = 0.0040) that can increase colonic mucus production to improve intestinal barrier function. Amino acid metabolites included cysteine (decreased 0.40-fold in LIC, *p* = 0.033, and 0.62-fold in serum, *p* = 0.014 of Pro+RB), which has been found to reduce inflammation, lower oxidative stress and modulate mucosal immunity, and histamine (decreased 0.18-fold in LIC, *p* = 0.00030, of Pro+RB and 1.57-fold in serum, *p* = 0.043), which modulates local and systemic inflammatory responses as well as influences the enteric nervous system. Alterations to entire LIC and serum metabolic pathways further contributed to the anti-diarrheal and anti-viral activities of Pro+RB such as sphingolipid, mono/diacylglycerol, fatty acid, secondary bile acid, and polyamine metabolism. Sphingolipid and long chain fatty acid profiles influenced the ability of HRV to both infect and replicate within cells, suggesting that Pro+RB created a protective lipid profile that interferes with HRV activity. Polyamines act on enterocyte calcium-sensing receptors to modulate intracellular calcium levels, and may directly interfere with rotavirus replication. These results support that multiple host and probiotic metabolic networks, notably those involving lipid and amino acid/peptide metabolism, are important mechanisms through which Pro+RB protected against HRV diarrhea in neonatal gnotobiotic pigs.

## Introduction

Globally, HRV is the most common cause of severe diarrhea in children under 5, and annually it is responsible for approximately 450,000 deaths worldwide (Clarke and Desselberger, 2015). Live attenuated vaccines, including RV5 and Rotarix, have greatly reduced overall mortality and gastroenteritis in many international vaccine programs, yet they have a markedly lower efficacy in developing countries where most HRV outbreaks still occur (Yen et al., 2014). Underlying pathological processes in the host intestinal tract may contribute to this reduced HRV vaccine efficacy and include environmental enteric dysfunction, where abnormal intestinal barrier and mucosal immune functions result primarily or secondarily from dysbiosis of the microbiome (Keusch et al., 2016). The combination of limited vaccine efficacy and compromised gut function warrants the need for more effective preventive strategies against HRV, especially in developing countries.

Probiotic bacteria, including LGG and EcN, represent a safe, alternative therapeutic that can reduce the severity of HRV diarrhea. In humans and animals, probiotics modulate mucosal immunity, reduced HRV binding and infection, and produced antimicrobial peptides with anti-viral activity against HRV (Chenoll et al., 2016; Olaya Galan et al., 2016; Vlasova et al., 2016; Wang et al., 2016). In gnotobiotic pigs, LGG demonstrated a strong adjuvant effect when supplemented with an oral attenuated HRV vaccine and increased mucosal populations of HRV-specific IFN-gamma producing T lymphocytes (Wen et al., 2015). Collectively, these results support that probiotics can function as an effective component of a prophylactic HRV treatment.

The ability of probiotics to combat HRV can be enhanced when they are combined into a synbiotic with appropriate prebiotics, which are exclusive nutrient sources for probiotic bacteria that cannot be digested by the host. RB, the outer covering of the rice grain, represents an affordable, sustainable, and globally produced source of prebiotics. Prior research demonstrated that gnotobiotic neonatal pigs fed a prophylactic synbiotic combination of LGG/EcN and RB (Pro+RB) did not experience diarrhea after oral challenge with a high dose of live, virulent HRV, whereas pigs consuming LGG/EcN alone (Pro) or RB showed only reductions in diarrhea (Yang et al., 2015). In piglets consuming Pro+RB, diarrhea elimination was associated with normal tissue histology and healthy levels of serum gut permeability markers throughout the HRV challenge (Yang et al., 2015). Additional studies with this pig model have demonstrated the ability of Pro+RB to substantially reduce human norovirus-associated diarrhea, while stimulating more potent adaptive immune responses against viral antigens, and preserving colonic tissue architecture during infection (Lei et al., 2016). These findings suggested that the synbiotic combination of a probiotic and a prebiotic functioned through multiple immune, gut barrier protective and anti-diarrheal mechanisms to enhance protection against HRV diarrhea. The increased efficacy may arise in part through RB modulations of probiotic metabolism (Henderson et al., 2012).

Metabolomics, the systematic study of metabolites, metabolic pathways, and their interconnected networks, can be utilized to evaluate how RB alters probiotic function to prevent diarrhea. The objective of this study was to compare the LIC and serum metabolome of pigs consuming a prophylactic combination of Pro+RB to those consuming probiotics alone. This metabolic fingerprint shows how alterations to metabolic networks and pathways function to protect the host against HRV diarrhea. The hypothesis is that the LIC and serum metabolome of neonatal gnotobiotic pigs consuming Pro+RB contains pathways and networks of metabolites associated with enhanced anti-diarrheal and gut mucosal immune-modulatory activities.

## Materials and Methods

### Experimental Design and Sample Collection

**FIGURE 1:**
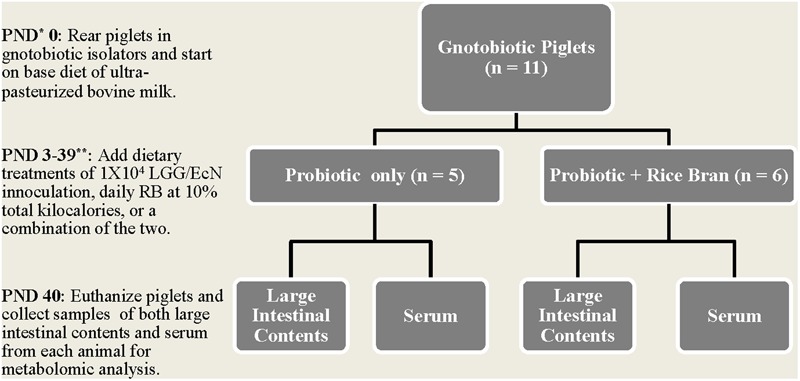
**Experimental design and sample collection.** ^∗^PND indicates postnatal day. ^∗∗^LGG/EcN refers to a 1:1 cocktail of *Lactobacillus rhamnosus* GG/*Escherichia coli Nissle*, which was provided to the appropriate treatment groups on postnatal days 3, 5, and 7. RB refers to Calrose rice bran.

Neonatal piglets (Large White crossbred) were reared in gnotobiotic isolators and maintained on experimental treatments as described previously by Yang et al. (2015). Briefly, piglets were started on a diet of ultra-pasteurized bovine milk, and from PND 3 through 40, the animals were separated into dietary treatments. Piglets in Pro group were inoculated with a sub-therapeutic dosage of 1 × 10^4^ LGG/EcN on PND 3, 5, and 7 to ensure adequate colonization of the intestinal tract. Animals in the Pro+RB treatment group were also fed heat-stabilized Calrose RB daily at 10% of their caloric intake. On PND 40, piglets were euthanized and LIC and serum samples were collected from each animal. The experimental design, including a time course for sample collection, is included as **Figure 1**. All animal care and use procedures were approved by the Institutional Animal Care and Use Committee at Virginia Polytechnic Institute and State University and all ample collection and experimental procedures were conducted with accordance to the approved guidelines.

### Metabolome Sample Preparation

To evaluate differences between Pro+RB and Pro treatments, a metabolomic analysis was performed by Metabolon Inc. © (Durham, NC, USA). Briefly, 0.5 mL of LIC or serum were collected in triplicate from each animal, stored at -80°C in microcentrifuge tubes, and were shipped to Metabolon on dry ice. Samples were stored at -80°C until processing. Prior to metabolite detection, a methanol solution was added to each sample to extract proteins and improve the recovery of small molecules. Samples were vigorously shaken for 2 min and then centrifuged. The resulting extracts were split into five parts for analysis via UPLC-MS/MS: two replicates for reverse phase UPLC-MS/MS using positive ion mode electrospray ionization, a third replicate for reverse phase UPLC-MS/MS with negative ion mode electrospray ionization, a fourth replicate for HPLC-MS/MS negative ion mode electrospray ionization, and a fifth replicate saved as a back-up. To remove any remaining organic solvent from the fractionation, extracts were placed on a TurboVap © (Zymark), and were then stored in liquid nitrogen prior to analysis.

### UPLC-MS/MS Analysis

Metabolite detection was accomplished using a Waters ACQUITY UPLC, a Thermo Scientific Q-exactive heated electrospray ionization source, and an Orbitrap mass analyzer operated at a 35,000 mass resolution. Each extract was dried and reconstituted in solvents appropriate for each detection method. Internal standards were added for quality control purposes. Acidic positive ion mode replicates were optimized for detection of either hydrophobic or hydrophilic compounds by gradient eluting in a mobile phase of water, methanol and 0.05% perfluoropentanoic acid and 0.1% formic acid (hydrophilic compounds), or methanol, acetonitrile, water, 0.05% perfluoropentanoic acid and 0.01% formic acid. All acidic positive ion mode replicates were ran on a C18 column (Waters UPLC BEH C100 18-2.1 mm × 150 mm, 1.7 μm). One basic ion extracts were eluted using an identical, separate C18 column with methanol, water, and 6.5 mM of Ammonium Bicarbonate at pH 8. The fourth extract was eluted using a high-performance liquid chromatography column (Waters UPLC BEH Amide 2.1 mm × 150 mm, 1.7 μm) with water, acetonitrile and 10 mM ammonium formate at pH 10.8. Mass spectrometry was performed using MS and data-dependent MSn scans using dynamic exclusion, and the total scan range covered 70–10,000 m/z.

### Data Extraction and Compound Identification

Raw data was extracted, peak-identified and quality-controlled as described previously (Brown et al., 2016). Briefly, compound identifications were compared to an internal library of over 3300 purified standards or recurrent unknown entities and were matched to library entities based on a narrow retention time/index, a mass to charge ratio within 10 parts per million of the standard, and chromatographic data including MS/MS forward and reverse scores between the experimental data and authentic standards. The raw counts of each sample were quantified using area under the curve and converted into relative abundances which were median-scaled to one. For each metabolite, fold difference was calculated by dividing the scaled relative abundance of Pro+RB by Pro.

### Metabolic Pathway Analysis

Identified metabolites were organized into metabolic pathways based on their biochemical and physiological properties. Pathway enrichment scores were calculated by dividing the number of significant metabolites in a pathway **(k)** by the total number of detected metabolites in the pathway **(m)**. This value was then divided by the fraction of the total number of significant metabolites in the data set **(n)** over the total number of detected metabolites in the complete dataset **(N)**:

$$\frac{k/m}{n/N}$$

Metabolic pathways with enrichment scores greater than one indicated that the pathway contained more metabolites with statistically significant fold differences compared to all other pathways within the matrix. In this investigation, there were 20 LIC and 16 serum pathways with a score greater than one. An additional layer to score interpretation was applied such that pathways containing only metabolites with established functions related to anti-diarrheal activity were selected for significance in results interpretations and further network analysis.

For both LIC and serum lipid metabolites, pathway enrichment scores were calculated for the following pathways: steroid, lysolipid and lysoplasmalogen, acyl choline, glycine and carnitine, phospholipid, dicarboxylate fatty acid, mevalonate, endocannabinoid, primary bile acid, medium chain fatty acid, amide and branched fatty acid, mono/dihydroxy fatty acid, sterols, polyunsaturated (n3 and n6) fatty acid, long chain fatty acid, secondary bile acid, mono/diacylglycerol, sphingolipid, ketone and glycerolipid. For LIC and serum amino acids/peptides, pathway enrichment scores were calculated for the following pathways: leucine isoleucine and valine, tryptophan, dipeptide and dipeptide derivative, phenylalanine and tyrosine, glycine, serine and threonine, urea, arginine and proline, lysine, glutamate, methionine, cysteine, taurine and *S*-adenosyl methionine, glutathione, polyamine, histidine, creatine, alanine and aspartate, gamma-glutamyl amino acid, and guanidino and acetamido. Pathway enrichment scores were visualized using GraphPad Prism 6.07 (San Diego, CA, USA).

### Metabolic Network Analysis

Pathways containing metabolites with established functions related to anti-diarrheal activity were selected for visualization using Cytoscape 2.8.3 © software as previously described (Brown et al., 2016). Visualization of Pro+RB versus Pro included all metabolites from selected lipid and amino acid/peptide pathways in either the LIC or serum matrix. Node diameter was determined by the magnitude of the fold difference between Pro+RB and Pro, and node color was determined by the direction of fold difference when comparing Pro+RB and Pro. Pathway enrichment scores are presented as a number on each pathway node. The LIC lipid pathways visualized in Cytoscape were: phospholipid, sphingolipid, mono/diacylglycerol, polyunsaturated (n3 and n6) fatty acid, endocannabinoid, sterol, long chain fatty acid, mevalonate, medium chain fatty acid, dihydroxy fatty acid, primary bile acid, and secondary bile acid. A visual of the serum lipid pathways included: mono/dihydroxy fatty acid, monoacylglycerol, endocannabinoid, phospholipid, and dicarboxylate fatty acid. A visual of the LIC amino acid/peptide pathways included: glutamate, histidine, urea, arginine and proline, tryptophan, polyamine, alanine and aspartate, methionine, cysteine, *S*-adenosyl methionine and taurine, carnitine, dipeptide/derivatives, and gamma-glutamyl amino acid. A visual of the serum amino acid/peptide pathways included: guanidino and acetamido, creatine, histidine, glutamate, urea, arginine and proline, and methionine, cysteine, taurine and *S*-adenosyl methionine.

### Statistical Analysis

Statistical analysis was performed by Metabolon Inc. © using ArrayStudio (Omicsoft, Cary, NC, USA). Briefly, the raw abundance of each metabolite from each sample of Pro or Pro+RB was median-scaled into a relative abundance. In both the LIC and serum, the contrast of Pro+RB versus Pro was selected for analysis from a larger, multi-treatment data set where a 2-way ANOVA was applied across all treatments (data not shown). Specifically, scaled metabolite abundance values were compared between Pro+RB and Pro using a Welch’s two-sample *t*-test. Statistical significance was determined at the level of *p* < 0.05. Due to the multiple comparisons inherent to the 2-way ANOVA analysis, a false discovery rate (q) was calculated for each metabolite

## Results

### Non-targeted, Global Metabolomics Revealed Differences between Probiotic+Rice Bran and Probiotic Treatments

Across Pro+RB and Pro treatments, 625 total metabolites were detected in the LIC and 532 metabolites were detected in the serum (**Table 1**). Seven hundred forty-six unique metabolites were detected across both matrices, where 415 were shared between LIC and serum, 214 were exclusive to the LIC, and 117 were exclusive to the serum. Of the 625 LIC metabolites, 170 were classified as amino acids, 31 peptides, 30 carbohydrates, 10 TCA cycle, 238 lipids, 39 nucleotides, 32 cofactors and vitamins, 54 phytochemicals/other. Of the 532 serum metabolites, 153 were classified as amino acids, 22 peptides, 21 carbohydrates, 9 TCA cycle, 229 lipids, 39 nucleotides, 25 cofactors and vitamins, and 32 phytochemicals.

**Table 1:** Number of identified metabolites from large intestinal contents and serum for pigs consuming probiotics in the presence and absence of rice bran (*p* < 0.05).

Metabolite classification	Large intestinal contents	Serum
Amino acids	170^∗^ (↑7, ↓78)^∗∗^	153 (↑14, ↓15)
Peptides	31 (↑2, ↓14)	24 (↑0, ↓2)
Carbohydrates	32 (↑2, ↓11)	21 (↑0, ↓1)
Energy metabolism	10 (↑0, ↓3)	9 (↑0, ↓0)
Lipids	257 (↑47, ↓58)	229 (↑15, ↓16)
Nucleotides	39 (↑1, ↓14)	39 (↑6, ↓1)
Cofactors and vitamins	32 (↑4,↓16)	25 (↑8, ↓0)
Phytochemical/Other	54 (↑15, ↓21)	32 (↑3, ↓3)
Total number of identified metabolites	625 (293)	532 (84)

*^∗^Indicates the total number of metabolites detected in a given metabolite class. ^∗∗^Values in parentheses indicate the number of significantly different (*p* < 0.05) metabolites that increased (**↑**) or decreased (↓) when comparing the fold difference of its scaled relative abundance in Pro+RB by Pro.*

There were 293 LIC metabolites and 84 serum metabolites with statistically significant (*p* < 0.05) fold differences when comparing Pro+RB to Pro (**Table 1**). In the LIC, 78 metabolites had a significantly higher scaled relative abundance in Pro+RB compared to Pro and included: 7 amino acids, 2 peptides, 2 carbohydrates, 47 lipids, 1 nucleotide, 4 cofactors/vitamins, and 15 phytochemical/other. Two hundred fifteen LIC metabolites had a significantly lower scaled relative abundance in Pro+RB compared to Pro and included: 78 amino acids, 14 peptides, 11 carbohydrates, 3 TCA cycle, 58 lipids, 14 nucleotides, 16 cofactors/vitamins, and 21 phytochemical/other.

In the serum, 45 metabolites had a significantly higher scaled relative abundance in Pro+RB compared to Pro and included: 14 amino acids, 15 lipids, 6 nucleotides, 8 cofactors/vitamins, and 3 phytochemical/other. Thirty-eight metabolites had a significantly lower scaled relative abundance in Pro+RB compared to Pro and included: 15 amino acids, 1 peptide, 1 carbohydrate, 17 lipids, 1 nucleotide, and 1 cofactor/vitamin.

### Lipid Metabolite Profiles Were Differentially Expressed between Probiotic+Rice Bran and Probiotic Treatment Groups and Were Associated with Anti-diarrheal Activity

Across both the LIC and serum, lipids represented ∼41 and 43% of the identified metabolites, respectively, and thus were examined more closely for their prospective anti-diarrheal-related properties. Supplementary Table 1 lists the significant (*p* < 0.05) lipids with the fold difference and *p*-values provided across both LIC and serum matrices, and Supplementary Table 3 presents a comprehensive literature search into the anti-diarrheal functions the lipids with statistically significant fold differences between Pro+RB and Pro. Analysis revealed 26 metabolites that possessed immunomodulatory, gut barrier protective and anti-viral functions related to anti-diarrheal activity. In the LIC, these lipids included long chain fatty acid (12 significant metabolites), polyunsaturated fatty acid (8 significant metabolites), phospholipid (5 significant metabolites), mono/diacylglycerol (14 significant metabolites), endocannabinoid (3 significant metabolites), sterol (4 significant metabolites), mevalonate (1 significant metabolite), primary bile acid (5 significant metabolites), secondary bile acid (7 significant metabolites), dihydroxy fatty acid (2 significant metabolites), medium chain fatty acid (3 significant metabolites), and sphingolipids (16 significant metabolites). In the serum, these lipids included mono/dihydroxy fatty acid (5 significant metabolites), endocannabinoid (1 significant metabolite), phospholipid (8 significant metabolites), and dicarboxylate fatty acid (5 significant metabolites).

### Amino Acid and Peptide Metabolite Profiles Differ between Probiotic+Rice Bran and Probiotic Treatment Groups and Are Associated with Anti-diarrheal Activity

The LIC and serum amino acid/peptide metabolites represented ∼27 and 28% of the total number of identified metabolites, respectively, and like lipids, were examined more closely for compounds with potential anti-diarrheal activity. Supplementary Table 2 lists the amino acid/peptide metabolites with statistically significant (*p* < 0.05) fold differences and *p*-values between Pro+RB and Pro across both LIC and serum matrices. Supplementary Table 4 presents a comprehensive literature search of the anti-diarrheal functions of amino acids/peptides with statistically significant fold differences between Pro+RB and Pro. Analysis revealed 22 metabolites that possessed immunomodulatory, gut barrier protective and anti-viral functions related to anti-diarrheal activity (Supplementary Table 4). In the LIC these amino acids/peptides included glutamate (6 significantly different metabolites), histidine (10 significantly different metabolites), urea cycle arginine and proline (10 significantly different metabolites), tryptophan (5 significantly different metabolites), polyamine (6 significantly different metabolites), alanine and aspartate (6 significantly different metabolites), methionine, cysteine, SAM, and taurine (11 significantly different metabolites), dipeptide/derivatives (6 significantly different metabolites), gamma-glutamyl amino acid (10 significantly different metabolites), and carnitine (2 significantly different metabolites). In the serum, these amino acid/peptide metabolites included urea cycle, arginine, and proline (5 significantly different metabolites), phenylalanine and tyrosine (2 significantly different metabolites), methionine, cysteine, SAM, and taurine (4 significantly different metabolites), histidine (6 significantly different metabolites), and glutamate (3 significantly different metabolites).

### Metabolic Pathways in the LIC and Serum Containing Anti-diarrheal Metabolites Were Associated with High Pathway Enrichment Scores

**FIGURE 2:**
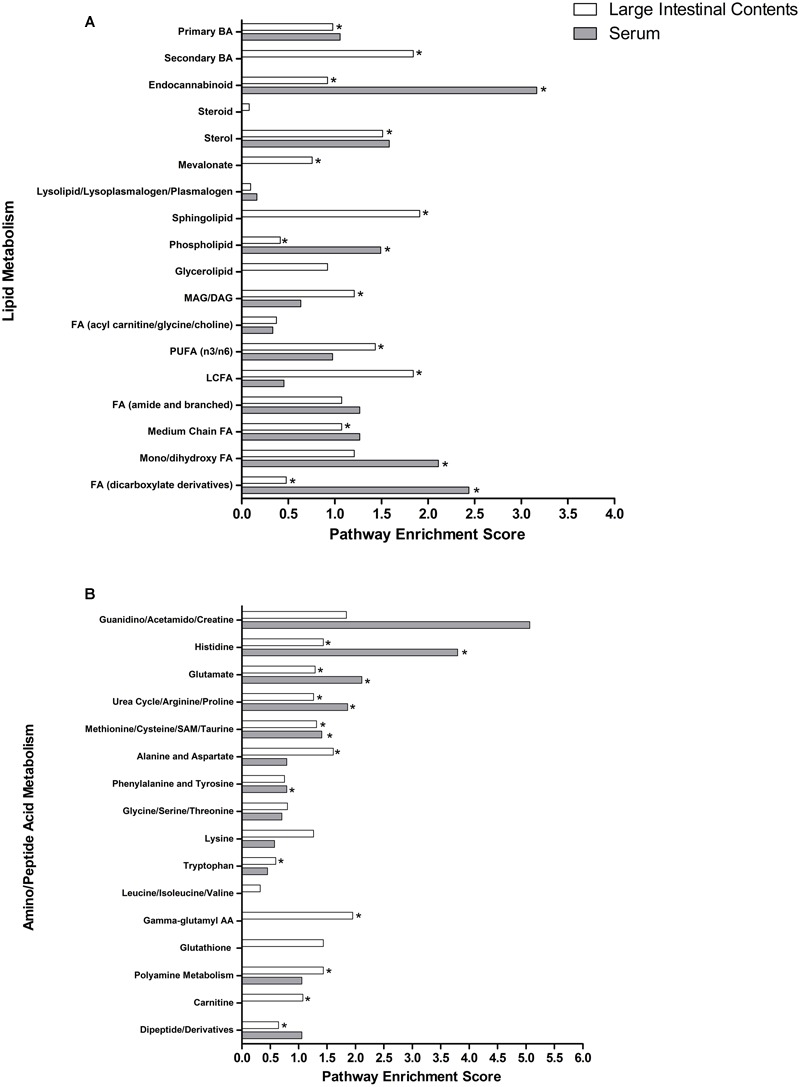
**Lipid and amino acid/peptide pathway enrichment scores in the large intestinal contents and serum when comparing Probiotic + Rice Bran versus Probiotic.** Pathway enrichment scores of **(A)** lipid pathways and **(B)** amino acid/peptide pathways are plotted for both large intestinal (white bars) and serum matrices (black bars). ^∗^ Indicates a pathway containing metabolites identified as having anti-diarrheal activity. In **(A)**, FA indicates fatty acid, BA indicates bile acid, MAG/DAG indicates monoacyl/diacylglycerol, PUFA indicates polyunsaturated fatty acid, and LCFA indicates long chain fatty acid. In **(B)**, SAM indicates *S*-adenosyl methionine, and AA indicates amino acid.

To examine how individual metabolic pathways contributed to Pro+RB versus Pro differences in protection from HRV diarrhea in pigs, pathway enrichment scores were calculated for each individual lipid (**Figure 2A**) and amino acid/peptide (**Figure 2B**) pathway in the LIC and serum as described in the methods. In general, many of these pathways exhibited a high pathway enrichment score (>1.00), supporting that these pathways are major contributors to treatment differences between Pro+RB and Pro. Lipid pathways in the LIC that contained anti-diarrheal metabolites included: phospholipid (score 0.41), sphingolipid (score 1.90), mono/diacylglycerol (score 1.20), polyunsaturated fatty acid (score 1.43), endocannabinoid (score 0.92), sterol (score 1.51), long chain fatty acid (score 1.84), mevalonate (score 0.75), medium chain fatty acid (score 1.07), mono/dihydroxy fatty acid (score 1.20), primary bile acid (score 0.97), and secondary bile acid (score 1.84). In the serum, lipid metabolic pathways containing anti-diarrheal metabolites included mono/dihydroxy fatty acid (score 2.11), endocannabinoid (score 3.16), phospholipid (score 1.49), dicarboxylate fatty acid (score 2.43). For lipids, metabolic pathways that contained anti-diarrheal metabolites in both the LIC and serum matrices included dicarboxylate fatty acid, endocannabinoid, phospholipid, and mono/dihydroxy fatty acid.

Amino acid/peptide metabolic pathways in the LIC containing anti-diarrheal metabolites included: glutamate (score 1.28), histidine (score 1.43), urea cycle, arginine, and proline (score 1.26), tryptophan (score 0.59), polyamine (score 1.43), alanine and aspartate (score 1.61), methionine, cysteine, SAM, and taurine (score 1.31), dipeptide/derivatives (score 0.64), gamma-glutamyl amino acids (score 1.95), and carnitine (score 1.07). In the serum, amino acid/peptide metabolic pathways containing anti-diarrheal metabolites included: urea cycle, arginine, and proline (score 1.86), phenylalanine and tyrosine (score 0.79), methionine, cysteine, SAM, and taurine (score 1.40), histidine (score 3.80), and glutamate (score 2.11). For amino acids, metabolic pathways that contained anti-diarrheal metabolites in both the LIC and serum matrices included histidine, glutamate, urea, arginine and proline, and methionine (**Figure 2B**).

### Cytoscape Analysis Reveals Network-wide Alterations in Lipid and Amino Acid Metabolite Profiles Associated with Anti-diarrheal Activity

**FIGURE 3:**
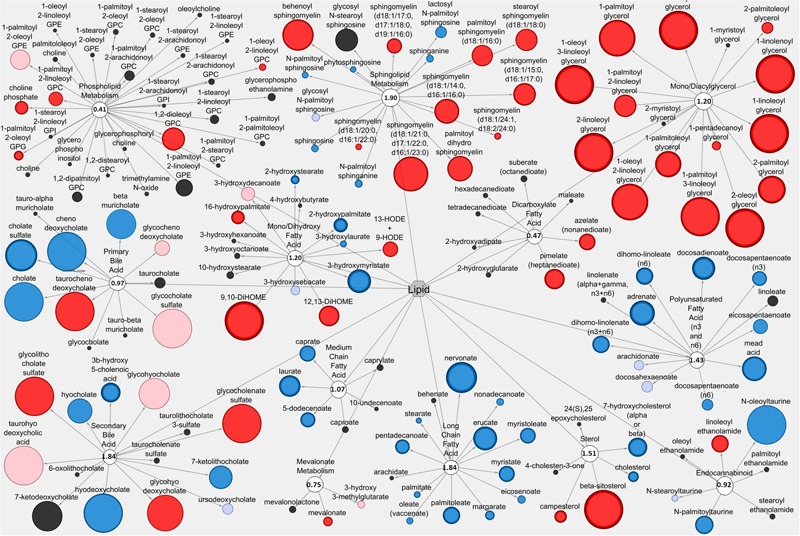
**Cytoscape network analysis of select lipid pathways in the large intestinal contents when comparing Probiotic + Rice Bran and Probiotic.** Cytoscape visualization of lipid metabolites in LIC metabolic pathways associated with anti-diarrheal activity across Pro+RB and Pro. For each metabolite, node diameter is proportional to the magnitude of the fold difference in Pro+RB compared to Pro. Node color indicates the direction of a metabolite’s fold difference: red indicates metabolites with a higher scaled abundance in Pro+RB (*p* < 0.05), blue indicates lower abundance in Pro+RB (*p* < 0.05), pink indicates trending higher in Pro+RB (0.05 < *p* < 0.10), and light blue indicates trending lower in Pro+RB (0.05 < *p* < 0.10). Black nodes indicate metabolites with fold differences that were not significantly altered between treatments. The pathway enrichment score is the number in the circle for each pathway.

**FIGURE 4:**
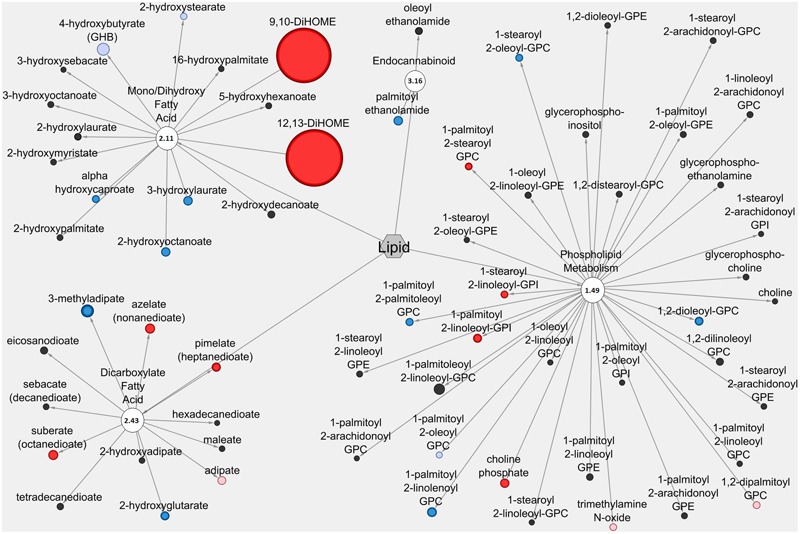
**Cytoscape network analysis of select lipid pathways in the serum when comparing Probiotic + Rice Bran and Probiotic.** Cytoscape visualization of lipid metabolites in serum metabolic pathways associated with anti-diarrheal activity across Pro+RB and Pro. For each metabolite, node diameter is proportional to the magnitude of the fold difference in Pro+RB compared to Pro. Node color indicates the direction of a metabolite’s fold difference: red indicates metabolites with a higher scaled abundance in Pro+RB (*p* < 0.05), blue indicates lower abundance in Pro+RB (*p* < 0.05), pink indicates trending higher in Pro+RB (0.05 < *p* < 0.10), and light blue indicates trending lower in Pro+RB (0.05 < *p* < 0.10). Black nodes indicate metabolites with fold differences that were not significantly altered between treatments. The pathway enrichment score is the number in the circle for each pathway.

The Cytoscape visualization tool classified individual metabolites into metabolic networks of their corresponding pathways. Lipid metabolites and pathways containing metabolites with anti-diarrheal activity were visualized in the LIC (**Figure 3**) and the serum (**Figure 4**). In the LIC, the lipid metabolic network displayed large increases in multiple lipid metabolites from Pro+RB when compared to Pro. Many of these increases corresponded to metabolites from mono/diacylglycerol, and sphingolipid pathways and included the anti-diarrheal associated metabolites 2-palmitoylglycerol (increased 7.08-fold in Pro+RB, *p* = 6.33E-05), 1-linoleoyl glycerol (increased 320.72-fold in Pro+RB, *p* = 1.04E-08), 2-linoleoylglycerol (increased 187.08-fold in Pro+RB, *p* = 9.27E-10), palmitoyl sphingomyelin (d18:1/16:0) (increased 5.42-fold in Pro+RB, *p* = 0.017), and sphingosine (decreased 0.29-fold in Pro+RB, *p* = 0.029) (**Figure 3**). There were LIC lipid pathways with large decreases in multiple lipid metabolites from Pro+RB when compared to Pro involving medium chain, long chain, and polyunsaturated fatty acids. Associated anti-diarrheal metabolites included stearate (decreased 0.65-fold in Pro+RB, *p* = 0.015), palmitate (decreased 0.59-fold in Pro+RB, *p* = 0.0087), oleate/vaccenate (decreased 0.56-fold in Pro+RB, *p* = 0.030), caprate (decreased 0.24-fold in Pro+RB, *p* = 0.0012), docaspentaenoate (22:5n3) (decreased 0.26-fold in Pro+RB, *p* = 0.032), eicosapentaenoate (20:5n3) (decreased 0.26-fold in Pro+RB, *p* = 0.041), and palmitoleate (16:1n7) (decreased 0.22-fold in Pro+RB, *p* = 0.00026) (**Figure 3**). In the serum, the lipid metabolic network displayed both large increases and decreases in lipid metabolites when Pro+RB was compared to Pro. Specifically, mono/dihydroxy and dicarboxylate fatty acid pathways exhibited large increases in the anti-diarrheal metabolites 12,13-dihydroxyoctadecenoic acid (increased 15.03-fold in Pro+RB, *p* = 0.00031), pimelate (increased 1.58-fold in Pro+RB, *p* = 0.010), and azelate (increased 1.73-fold in Pro+RB, *p* = 0.023) in Pro+RB compared to Pro. Phospholipid metabolism resulted in large decreases in 1-palmitoyl-2-palmitoleyol-glycerophosphocholine (decreased 0.71-fold in Pro+RB, *p* = 0.028), 1-palmitoyl-2-linoleyol glycerophosphocholine (decreased 0.59-fold in Pro+RB, *p* = 0.015), 1-stearoyl-2-oleoyl glycerophosphocholine (decreased 0.73-fold in Pro+RB, *p* = 0.045), and 1,2-dioleoyl glycerophosphocholine (decreased 0.65-fold in Pro+RB, *p* = 0.014) when comparing Pro+RB and Pro (**Figure 4**).

**FIGURE 5:**
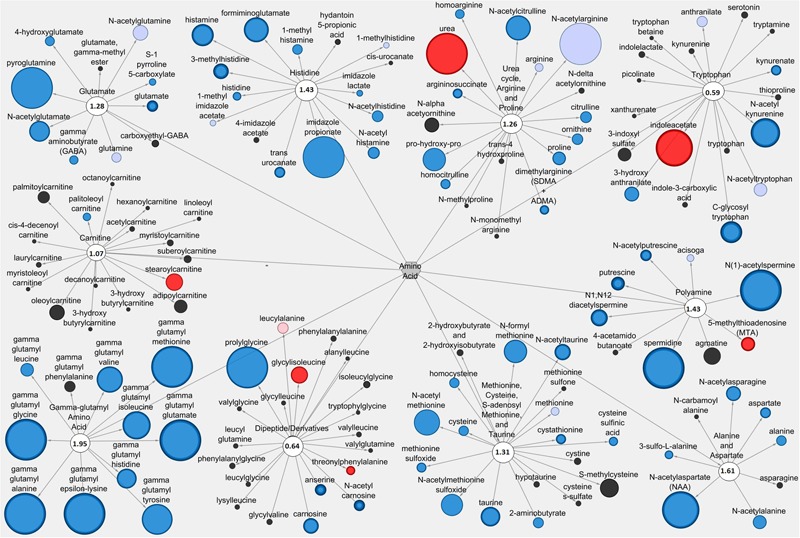
**Cytoscape network analysis of select amino acid/peptide pathways in the large intestinal contents when comparing Probiotic + Rice Bran and Probiotic.** Cytoscape visualization of amino acid/peptide metabolites in LIC metabolic pathways associated with anti-diarrheal activity across Pro+RB and Pro. For each metabolite, node diameter is proportional to the magnitude of the fold difference in Pro+RB compared to Pro. Node color indicates the direction of a metabolite’s fold difference: red indicates metabolites with a higher scaled abundance in Pro+RB (*p* < 0.05), blue indicates lower abundance in Pro+RB (*p* < 0.05), pink indicates trending higher in Pro+RB (0.05 < *p* < 0.10), and light blue indicates trending lower in Pro+RB (0.05 < *p* < 0.10). Black nodes indicate metabolites with fold differences that were not significantly altered between treatments. The pathway enrichment score is the number in the circle for each pathway.

**FIGURE 6:**
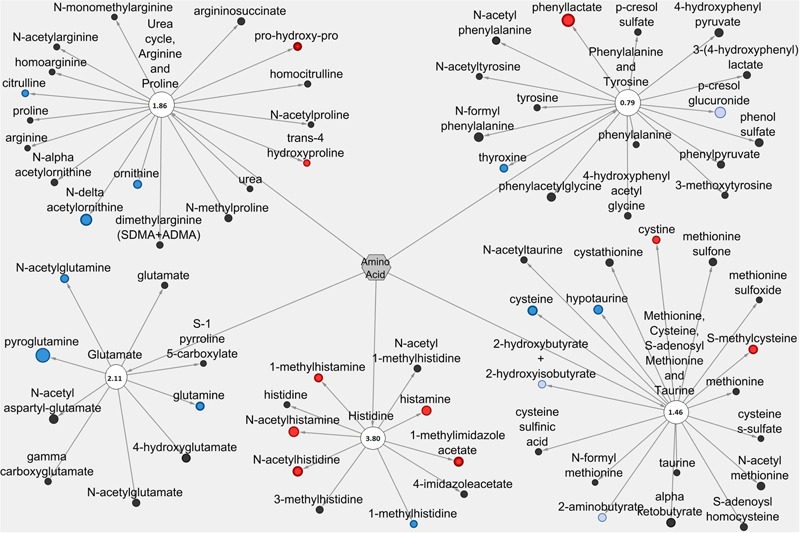
**Cytoscape network analysis of select amino acid/peptide pathways in the serum when comparing Probiotic + Rice Bran and Probiotic.** Cytoscape visualization of amino acid/peptide metabolites in serum metabolic pathways associated with anti-diarrheal activity across Pro+RB and Pro. For each metabolite, node diameter is proportional to the magnitude of the fold difference in Pro+RB compared to Pro. Node color indicates the direction of a metabolite’s fold difference: red indicates metabolites with a higher scaled abundance in Pro+RB (*p* < 0.05), blue indicates lower abundance in Pro+RB (*p* < 0.05), pink indicates trending higher in Pro+RB (0.05 < *p* < 0.10), and light blue indicates trending lower in Pro+RB (0.05 < *p* < 0.10). Black nodes indicate metabolites with fold differences that were not significantly altered between treatments. The pathway enrichment score is the number in the circle for each pathway.

**Figures 5**, **6** display amino acid/peptide LIC and serum metabolic pathways, respectively, containing anti-diarrheal-associated metabolites. In the LIC, Pro+RB displayed large decreases in multiple metabolites, across numerous pathways, when compared to Pro. These decreases corresponded to metabolites from gamma-glutamyl amino acid, polyamine, alanine and aspartate, histidine, glutamate, and methionine, cysteine, SAM, and taurine metabolism and included the anti-diarrheal associated metabolites gamma-glutamyl valine (decreased 0.15-fold in Pro+RB, *p* = 0.0011), gamma-glutamyl-epsilon-lysine (decreased 0.061-fold in Pro+RB, *p* = 2.18E-05), spermidine (decreased 0.038-fold in Pro+RB, *p* = 1.64E-10), alanine (decreased 0.40-fold in Pro+RB, *p* = 0.026), histidine (decreased 0.48-fold in Pro+RB, *p* = 0.012), histamine (decreased 0.18-fold in Pro+RB, *p* = 0.00030), 3-methylhistidine (decreased 0.26-fold in Pro+RB, *p* = 2.70E-05), gamma-aminobutyrate (GABA) (decreased 0.46-fold in Pro+RB, *p* = 0.023), glutamate (decreased 0.36-fold in Pro+RB, *p* = 2.38E-05), cysteine (decreased 0.40-fold, *p* = 0.033), homocysteine (decreased 0.41-fold in Pro+RB, *p* = 0.029), and taurine (decreased 0.21-fold in Pro+RB, *p* = 0.00034) (**Figure 5**). In the serum, amino acid/peptide metabolic network displayed both multiple increases in histidine metabolites of Pro+RB compared to Pro, including the anti-diarrheal associated metabolite histamine (increased 1.57-fold in Pro+RB, *p* = 0.043) (**Figure 6**).

## Discussion

Non-targeted global metabolomics revealed substantial alterations to both lipid and amino acid/peptide metabolism in the LIC and serum (**Table 1**), and multiple metabolite and metabolic pathway contributions to prophylactic diarrhea protection (**Figure 2** and Supplementary Tables 2, 3). This manuscript further highlights those compounds associated with protection against HRV diarrhea related to increased production of gut-protective, immunomodulatory, and anti-viral compounds.

Rice bran-mediated alterations to probiotic lipid metabolism represent one route through which a combination of probiotics and prebiotics create a gut-protective environment against HRV infection. RB provides probiotic bacteria with a source of complex lipids that can be catabolized to release additional lipids into the large intestinal lumen or converted into bacterial lipid products (Buccioni et al., 2012). Alterations to LIC mono/diacylglycerol metabolism (pathway enrichment score 1.20) represent one area of lipid metabolism associated with anti-diarrheal activity (**Figure 2**). Elevations of multiple mono/diacylglycerols in the LIC of Pro+RB included 1-linoleoyl glycerol (increased 320.72 fold in Pro+RB, *p* = 1.04E-08), 2-linoleoyl glycerol (increased 187.09 fold in Pro+RB, *p* = 9.27E-10), and 2-palmitoyl glycerol (increased 7.08 fold in Pro+RB, *p* = 6.33E-05) (Supplementary Tables 1, 3). 1-linoleoyl glycerol has been demonstrated to systemically reduce inflammation in porcine models (Han et al., 2015). 2-linoleoyl glycerol serves as an important precursor in the formation of 2-arachidonyl glycerol that can broadly modulate inflammation through direct binding to endocannabinoid receptors, or via its catabolism into ligands of cyclooxygenases, cytochrome P450 enzymes, and lipoxygenase enzymes (Mechoulam et al., 1995; Turcotte et al., 2015). Similarly, 2-palmitoylglycerol is produced by mammalian and bacterial cells and influences cannabinoid receptor expression in the gastrointestinal tract (Aviello et al., 2008; Murataeva et al., 2016; Yuan et al., 2016). Collectively, probiotic catabolism of complex RB triacylglycerides may release mono/diacylglycerols into the intestinal lumen and increase their bioavailability in the intestinal lumen, where they can be utilized in host enterocyte metabolism to exert their anti-diarrhea effects. Reducing inflammation along the gastrointestinal tract may provide the host with improved barrier function (De Jong et al., 2016; Lei et al., 2016), and modulating cannabinoid receptor function can alter intestinal motility (Taschler et al., 2017) that can help prevent them against HRV-induced diarrhea. Ultimately, these released mono/diacylglycerols could be utilized in host enterocyte metabolism and promote anti-inflammatory and anti-viral activities that protect the host from HRV diarrhea.

In a healthy gut, primary bile acids play key roles in facilitating the digestion and absorption of fats, nutrients, and vitamins (Martinot et al., 2017). A major function of probiotic bacteria is the bioconversion of host-derived primary bile acids into bacterially modified secondary bile acids which function as antimicrobials, regulate host lipid and glucose metabolism, and influence the growth and activity of gut flora (Nie et al., 2015). Analysis of LIC primary and secondary bile acid metabolites in Pro+RB versus Pro revealed anti-diarrheal activities of the primary bile acid chenodeoxycholate (decreased 0.054-fold in Pro+RB, *p* = 0.0078), and the secondary bile acid hyodeoxycholate (decreased 0.054-fold in Pro+RB, *p* = 0.0040) (Supplementary Tables 1, 3). Investigations have suggested that lower luminal levels of chenodeoxycholate are associated with decreased gastric emptying times and reduced expression of the pro-inflammatory cytokines TNF-a, IL-1B, IL-6, and IL-8 (Goyal et al., 2015; Peleman et al., 2016). The decrease in chenodeoxycholate may be associated with metabolism of hyodeoxycholate a secondary bile acid resulting from bacterial metabolism of chenodeoxycholate (Sabbatini et al., 2013; Theriot et al., 2016). In the colonic lumen, rodent studies suggest that hyodeoxycholate acts directly on goblet cells to increase mucus production, thereby modulating barrier function (Barcelo et al., 2001). RB supplementation may have increased probiotic conversion of chenodeoxycholate into hyodeoxycholate, which provided, that ultimately was taken-up by enterocytes, out of the lumen, and used to mediate anti-inflammatory processes in the cell, and thus improve barrier function that provided protection during challenge with HRV.

Probiotic production of fatty acids, along with bioconversion or release of RB fatty acids, may influence immune and metabolic processes that contribute to diarrheal protection. The dicarboxylic fatty acid pimelate was elevated in 5.17-fold the LIC (*p* = 0.00033) and 1.58-fold the serum (*p* = 0.01) of Pro+RB versus Pro (Supplementary Tables 1, 3) and is produced by a variety of bacterial species. Past investigations have demonstrated that pimelate produced by the probiotic *Bifidobacterium* spp. in mice can alter gut luminal metabolism by increasing the bioavailability of biotin to enterocytes, modulating the development of T-regulatory cells, and influencing the composition of the gut microbiome (Sugahara et al., 2015). In healthy human and animal enterocytes, biotin is an essential co-factor produced by gut flora and it has multiple roles in lipid and amino acid metabolism, including as a modulator of mucosal immune responses and gut microbial populations (Ghosal et al., 2015). Although not established in this gnotobiotic model of disease, HRV infections are thought to contribute to disruption of normal intestinal microflora (Zhang et al., 2014), suggesting HRV pathogenesis may indirectly alter host biotin metabolism. Pimelate may have enhanced biotin uptake into enterocytes to promote gut metabolic, microbial and immune homeostasis, thereby improving barrier function and reducing inflammation, and may have thus allowed the host to better resist diarrhea.

Rice bran modification to probiotic amino acid/peptide metabolism also provided prophylactic diarrhea protection. Probiotic bacteria can catabolize complex RB and host proteins to release smaller bioactive peptides and amino acids, or bioconvert plant and host amino acids into bacterial-derived amino acids (Neis et al., 2015; Pessione and Cirrincione, 2016) to create a novel profile of amino acids/peptides that participate in anti-diarrheal and anti-viral responses against HRV. Probiotic and host histidine metabolism was differentially modulated between Pro+RB and Pro groups. Histidine, which was decreased 0.12-fold in the LIC of Pro+RB when compared to Pro (*p* = 0.012) (Supplementary Tables 2, 4), functioned as an anti-secretory agent to reduce Cholera-associated diarrhea when supplemented into a rice-based oral rehydration salt (Rabbani et al., 2005), suggesting that it could function similarly to reduce the onset of HRV diarrhea.

Histamine, a downstream metabolite of histidine that can be produced by both animals and probiotics (Gao et al., 2015), decreased 0.19-fold in the LIC (*p* = 0.00030) and increased 1.57-fold in the serum (*p* = 0.043) of Pro+RB compared to Pro (Supplementary Tables 2, 4). Histamine is a well-characterized mediator of inflammation, mucosal immunity, and the enteric nervous system (Smolinska et al., 2014), yet it can suppress these processes when bound to specific receptors in the colon (Gao et al., 2015). Previous investigations with *Lactobacillus reuteri* demonstrated that probiotics can use a bacterial-derived histidine deactylase to convert dietary histidine into histamine in the colonic lumen, which acts on H2 receptors to suppress colonic inflammation and reduce damage to the colonic epithelium (Gao et al., 2015). Probiotics may have converted RB histidine into histamine that left the LIC to bind H2 receptors, caused local modulation of colonic inflammatory responses, and increased barrier function, all of which ultimately interfered with HRV pathogenesis and provided diarrhea protection.

Other contributors to osmotic balance and thus likely anti-diarrheal mediators include cysteine and cystine metabolites. Cysteine, decreased 0.40-fold in the LIC (*p* = 0.033) and 0.62-fold in the serum (*p* = 0.014) of Pro+RB compared to Pro (Supplementary Tables 2, 4), reduces oxidative stress, decreases inflammation, lowers permeability, and modulates the mucosal immune system in the intestinal tract of neonatal pigs (Bauchart-Thevret et al., 2009; Ruth and Field, 2013). Cystine, increased 1.30-fold (*p* = 0.044) in the serum of Pro+RB compared to Pro (Supplementary Tables 2, 4), serves as an excitatory neurotransmitter in the brainstem of humans and other animals, where it functions peripherally to delay gastric emptying and suppress food intake (Khan et al., 2014; McGavigan et al., 2015). During HRV infections, inflammation can cause enterocyte damage and increase gut permeability which both exacerbate HRV diarrhea (Yang et al., 2015). Prophylactic modulations to cysteine and cysteine induced by Pro+RB may favorably shape the mucosal immune response and enteric nervous system to protect enterocytes and gut flora against oxidative damage and maintain normal gut motility during HRV infections to diarrhea.

Pro+RB alterations to entire pathways, containing multiple metabolites working in synergy, have been theorized to have anti-diarrheal effects on HRV. This was most evident in the LIC lipid pathways including long chain fatty acid (pathway enrichment score 1.84), sphingolipid (score 1.90, LIC), and secondary bile acid (score 1.84, LIC) pathways (**Figures 2A, 3**), all of which contained multiple anti-diarrheal-associated metabolites and had pathway enrichment scores higher than one. This suggests that these pathways were contributors to treatment differences contributing to the anti-diarrheal enhancement of Pro+RB versus Pro.Systems of long chain fatty acids have been reported to influence HRV infectivity and replication in the intestinal tract (Kim et al., 2012), whereas pathway-wide shifts in sphingolipid metabolism can influence the composition of host membrane lipid rafts (Barenholz, 2004), where HRV must successfully bind to infect enterocytes. Similarly, entire profiles of secondary bile acids have been associated with reductions in HRV diarrhea in Kenyan children, and likely function to disrupt HRV function by altering the pH of the intestinal lumen (Tazume et al., 1990).

Entire amino acid/peptide pathways associated with anti-diarrheal function included gamma-glutamyl amino acid (pathway enrichment score 1.95, LIC) and polyamine metabolism (score 1.43, LIC). HRV diarrhea results in part due to impaired transport of gamma-glutamyl amino acids from the intestinal lumen and into enterocytes (Katyal et al., 1999). Large differences in the LIC gamma-glutamyl amino acid profile between Pro+RB and Pro (**Figures 2B, 5**) suggest that prophylactic alterations to luminal amino acid transport are associated with anti-diarrheal effects. In addition, HRV de-regulates host calcium metabolism and induces massive intracellular calcium increases to support HRV replication (Hyser et al., 2013). Pathway-wide modulation of LIC polyamine metabolites (**Figures 2B, 5**) in Pro+RB can work to reduce diarrhea and interfere with HRV replication. Past investigations support that polyamines in the intestinal lumen act on the enterocyte calcium-sensing receptor to inhibit the intracellular release of calcium and reduce chloride secretion into the intestinal lumen (Rogers et al., 2015), simultaneously interfering with rotavirus replication and altering luminal osmolarity to reduce the onset of diarrhea. Collectively, analysis of entire lipid and amino acid/peptide pathways supports that a more comprehensive understanding of anti-diarrheal activity can be achieved when clusters of closely related metabolites are considered as part of an entire, functioning metabolic system.

While many pathways appeared to be differentially regulated exclusively in the LIC or serum, multiple lipid and amino acid/peptide pathways contained anti-diarrheal-associated metabolites in both LIC and serum matrices. These pathways included dicarboxylate fatty acid, endocannabinoid, phospholipid, and mono/dihydroxy fatty acid, histidine, glutamate, urea, arginine and proline, and methionine. When coupled with their high pathway enrichment scores, this suggests that these pathways may be important both locally and systemically for promoting enhanced diarrheal protection in Pro+RB compared to Pro. For example, the glutamate metabolism (pathway enrichment score 1.28, LIC and 2.11 serum) (**Figure 2B**), works through the central and enteric nervous systems to modulate gut motility, intestinal fluid balance, and inflammation (Kondoh et al., 2009; Li et al., 2012; Auteri et al., 2015). In addition, endocannabinoid metabolism, (score 0.92 LIC and 3.16 serum) works locally in the intestinal lumen to improve barrier function, modulate motility, and improve mucosal integrity and systemically to influence immune-mediated inflammatory responses and central nervous system functioning that could ultimately influence the course of diarrheal diseases in the gastrointestinal tract (Goodgame, 1999; Lee et al., 2016; Gertsch, 2017).

Currently, variable vaccine efficacy and environmental enteric dysfunction exacerbate HRV outbreaks in the developing countries and warrant the development of stronger preventive strategies. The combination of probiotics and RB has the potential to serve as a safe, natural, prophylactic treatment aimed at reducing HRV diarrhea, and accomplishes this in part via multiple modulations to bacterial and host lipid and amino acid/peptide metabolism. Probiotic metabolism of RB can exert direct anti-viral activity against HRV by interfering with HRV infection, replication and pathogenesis, but also interfere with HRV pathogenicity by improving gut barrier function, and modulating mucosal immune responses, especially inflammatory processes that influence the course of HRV diarrhea. Direct modulations to host metabolism and immunity also indicate that while Pro+RB is effective against HRV, it is likely that this combination can provide broad protection against a wide range of pathogenic diarrheal diseases. Although the current investigation focused primarily on modulations to lipid and amino acid/peptide metabolism, there are likely other classes of metabolites that contribute to the anti-viral and anti-diarrheal enhancement associated with combinations of probiotics and RB. An evaluation of these other metabolic pathways can serve as future investigations into the molecular mechanisms of diarrhea protection by this combination. Probiotic and RB prophylactic therapies address a global need for more accessible, natural, antimicrobial agents, and have the potential to protect people and animals against a wide variety of enteric diseases.

## Author Contributions

LY, XY and ER designed animal studies, and LY and XY conducted animal studies. NN and ER performed metabolomic data analysis and wrote the manuscript. All authors approved the final manuscript for submission.

## Conflict of Interest Statement

The authors declare that the research was conducted in the absence of any commercial or financial relationships that could be construed as a potential conflict of interest.

## Abbreviations

EcN: *Escherichia coli* Nissle 1917
HPLC-MS/MS: high-performance liquid chromatography coupled with tandem mass spectrometry
HRV: human rotavirus
LGG: *Lactobacillus rhamnosus* GG
LIC: large intestinal contents
Pro: probiotic
Pro+RB: combination treatment of probiotic + rice bran
PND: postnatal day
RB: rice bran
UPLC-MS/MS: ultra-high performance liquid chromatography coupled with tandem mass spectrometry
