# Supplementary material for: Rice Bran and Probiotics Alter the Porcine Large Intestine and Serum Metabolomes for Protection against Human Rotavirus Diarrhea
Source: Front Microbiol. 2017 Apr 21;8:653. doi: 10.3389/fmicb.2017.00653 (PMC5399067; doi:10.3389/fmicb.2017.00653)
Supplement: Supplementary file 1 [file Table_1.DOCX]

Supplementary Material

Rice bran and Probiotics Alter the Porcine Large Intestinal and Serum Metabolomes for Enhanced Protection against Human Rotavirus Diarrhea

Nora Jean Nealon, Lijuan Yuan, Xingdong Yang, and Elizabeth P. Ryan*

*** Correspondence:** e.p.ryan@colostate.edu

**Supplementary Table 1. Large intestinal content and serum lipids in pigs consuming probiotics in the presence and absence of rice bran.**

| **Metabolite^*^** | **HMDB^**^** | **Large Intestinal Contents** | | **Serum** | |
| --- | --- | --- | --- | --- | --- |
|  |  | **Fold Difference^***^** | **p-value** | **Fold Difference** | **p-value** |
| 1-linoleoylglycerol (18:2) | - | 320.72 ↑ | 1.04E-08 | - | |
| 2-oleoylglycerol (18:1) | - | 293.40 ↑ | 3.04E-10 | - | |
| 2-linoleoylglycerol (18:2) | [11538](http://www.hmdb.ca/metabolites/HMDB11538) | 187.09 ↑ | 9.27E-10 | - | |
| 1-oleoyl-3-linoleoyl-glycerol (18:1/18:2) | - | 177.20 ↑ | 2.26E-08 | - | |
| 9,10-dihydroxyoctadecenoic acid (DiHOME) | [04704](http://www.hmdb.ca/metabolites/HMDB04704) | 111.38 ↑ | 1.90E-07 | 9.63 ↑ | 0.00016 |
| 1-oleoylglycerol (18:1) | [11567](http://www.hmdb.ca/metabolites/HMDB11567) | 93.63 ↑ | 3.37E-07 | - | |
| glycocholenate sulfate | - | 71.16 ↑ | 0.011 | - | |
| 1-linolenoylglycerol (18:3) | [11569](http://www.hmdb.ca/metabolites/HMDB11569) | 45.81 ↑ | 9.59E-07 | - | |
| taurochenodeoxycholate | [00951](http://www.hmdb.ca/metabolites/HMDB00951) | 16.34 ↑ | 0.012 | - | |
| beta-sitosterol | [00852](http://www.hmdb.ca/metabolites/HMDB00852) | 15.59 ↑ | 4.72E-11 | - | |
| glycolithocholate sulfate | [02639](http://www.hmdb.ca/metabolites/HMDB02639) | 12.09 ↑ | 0.0088 | - | |
| 1-palmitoyl-3-linoleoyl-glycerol (16:0/18:2) | - | 10.51 ↑ | 0.00013 | - | |
| 1-oleoyl-2-linoleoyl-glycerol (18:1/18:2) | - | 9.37 ↑ | 0.00021 | 1.79 ↑ | 0.033 |
| 1-palmitoylglycerol (16:0) | [31074](http://www.hmdb.ca/metabolites/HMDB31074) | 9.33 ↑ | 0.00014 | - | |
| glycohyodeoxycholate | - | 9.27 ↑ | 0.046 | - | |
| sphingomyelin (d18:1/21:0, d17:1/22:0, d16:1/23:0) | - | 8.89 ↑ | 0.0010 | - | |
| behenoyl sphingomyelin (d18:1/22:0) | - | 7.98 ↑ | 0.0012 | - | |
| 1-palmitoyl glycerophosphatidic acid (GPA) (16:0) | [00327](http://www.hmdb.ca/metabolites/HMDB00327) | 7.27 ↑ | 0.011 | - | |
| 2-palmitoylglycerol (16:0) | [11533](http://www.hmdb.ca/metabolites/HMDB11533) | 7.08 ↑ | 6.33E-05 | - | |
| 1-palmitoleoylglycerol (16:1) | - | 6.70 ↑ | 0.00015 | - | |
| palmitoyl dihydrosphingomyelin (d18:0/16:0) | - | 6.48 ↑ | 0.0028 | - | |
| sphingomyelin (d18:1/14:0, d16:1/16:0) | - | 6.33 ↑ | 0.0010 | - | |
| 1,2-dioleoyl-glycerophosphocholine (GPC) (18:1/18:1) | - | 5.88 ↑ | 0.0035 | 0.65 ↓ | 0.014 |
| sphingomyelin (d18:1/15:0, d16:1/17:0) | - | 5.75 ↑ | 0.0021 | - | |
| 1-palmitoyl-2-linoleoyl-glycerol (16:0/18:2) | 05207, 07103 | 5.71 ↑ | 0.0031 | - | |
| stearoyl sphingomyelin (d18:1/18:0) | [01348](http://www.hmdb.ca/metabolites/HMDB01348) | 5.61 ↑ | 0.0074 | - | |
| palmitoyl sphingomyelin (d18:1/16:0) | - | 5.42 ↑ | 0.017 | - | |
| 12,13-dihydroxyoctadecenoic acid (DiHOME) | [04705](http://www.hmdb.ca/metabolites/HMDB04705) | 5.26 ↑ | 0.0012 | 15.03 ↑ | 0.00031 |
| heptanedioate (pimelate) | 00857 | 5.17 ↑ | 0.00033 | 1.58 ↑ | 0.010 |
| malonate | [00691](http://www.hmdb.ca/metabolites/HMDB00691) | 4.81 ↑ | 1.26E-06 | - | |
| linoleoyl ethanolamide | [12252](http://www.hmdb.ca/metabolites/HMDB12252) | 4.42 ↑ | 0.0014 | - | |
| glycerol | [00131](http://www.hmdb.ca/metabolites/HMDB00131) | 4.32 ↑ | 0.00088 | - | |
| nonanedioate (azelate) | 00784 | 4.23 ↑ | 0.0015 | 1.73 ↑ | 0.023 |
| stearoylcarnitine | [00848](http://www.hmdb.ca/metabolites/HMDB00848) | 4.13 ↑ | 0.044 | - | |
| 13 + 9 Hydroxyoctadecadienoic acid (13+9 HODE) | - | 3.93 ↑ | 0.010 | - | |
| sphingomyelin (d18:1/17:0, d17:1/18:0, d19:1/16:0) | - | 3.88 ↑ | 0.0059 | - | |
| 1-palmitoyl-2-linoleoyl-glycerophosphocholine (GPC) (16:0/18:2) | - | 3.66 ↑ | 0.039 | - | |
| 16-hydroxypalmitate | 06294 | 3.45 ↑ | 0.00037 | - | |
| choline phosphate | 01565 | 3.43 ↑ | 0.0027 | 1.61 ↑ | 0.046 |
| campesterol | [02869](http://www.hmdb.ca/metabolites/HMDB02869) | 3.17 ↑ | 0.00047 | 10.69 ↑ | 1.58E-06 |
| 2-palmitoleoylglycerol (16:1) | - | 3.00 ↑ | 0.015 | - | |
| 1-palmitoyl-2-oleoyl-glycerophosphoglycerol (GPG) (16:0/18:1) | - | 2.47 ↑ | 0.0023 | - | |
| mevalonate | [00227](http://www.hmdb.ca/metabolites/HMDB00227) | 2.44 ↑ | 0.013 | - | |
| 1-pentadecanoylglycerol (15:0) | - | 2.34 ↑ | 0.0074 | - | |
| 1-oleoyl-2-linoleoyl-glycerophosphocholine (GPC) (18:1/18:2) | - | 1.84 ↑ | 0.033 | - | |
| sphingomyelin (d18:1/24:1, d18:2/24:0) | - | 1.77 ↑ | 0.0079 | - | |
| sphingomyelin (d18:1/20:0, d16:1/22:0) | - | 1.65 ↑ | 0.032 | - | |
| stearate (18:0) | [00827](http://www.hmdb.ca/metabolites/HMDB00827) | 0.65 ↓ | 0.015 | - | |
| phytosphingosine | [04610](http://www.hmdb.ca/metabolites/HMDB04610) | 0.63 ↓ | 0.034 | - | |
| palmitate (16:0) | [00220](http://www.hmdb.ca/metabolites/HMDB00220) | 0.59 ↓ | 0.0087 | - | |
| N-palmitoyl-sphingosine (d18:1/16:0) | [04949](http://www.hmdb.ca/metabolites/HMDB04949) | 0.59 ↓ | 0.025 | - | |
| 3-hydroxylaurate | 00387 | 0.58 ↓ | 0.014 | 0.60 ↓ | 0.046 |
| oleate (vaccenate) (18:1) | - | 0.56 ↓ | 0.030 | - | |
| sphingosine | [00252](http://www.hmdb.ca/metabolites/HMDB00252) | 0.53 ↓ | 0.029 | - | |
| nonadecanoate (19:0) | [00772](http://www.hmdb.ca/metabolites/HMDB00772) | 0.52 ↓ | 0.022 | - | |
| sphinganine | [00269](http://www.hmdb.ca/metabolites/HMDB00269) | 0.52 ↓ | 0.034 | - | |
| N-palmitoyl-sphinganine (d18:0/16:0) | [11760](http://www.hmdb.ca/metabolites/HMDB11760) | 0.50 ↓ | 0.0070 | - | |
| palmitoleoylcarnitine | - | 0.50 ↓ | 0.042 | - | |
| eicosenoate (20:1) | [02231](http://www.hmdb.ca/metabolites/HMDB02231) | 0.47 ↓ | 0.024 | - | |
| 2-hydroxystearate | - | 0.44 ↓ | 0.00034 | - | |
| margarate (17:0) | [02259](http://www.hmdb.ca/metabolites/HMDB02259) | 0.41 ↓ | 0.0062 | - | |
| 17-methylstearate | - | 0.40 ↓ | 0.0038 | - | |
| cholesterol | [00067](http://www.hmdb.ca/metabolites/HMDB00067) | 0.36 ↓ | 0.00052 | - | |
| docosapentaenoate (n6 DPA; 22:5n6) | 01976 | 0.34 ↓ | 0.032 | - | |
| lactosyl-N-palmitoyl-sphingosine | - | 0.33 ↓ | 0.041 | - | |
| 2-methylmalonyl carnitine | [13133](http://www.hmdb.ca/metabolites/HMDB13133) | 0.29 ↓ | 0.0012 | 1.52 ↑ | 0.034 |
| malonylcarnitine | [02095](http://www.hmdb.ca/metabolites/HMDB02095) | 0.28 ↓ | 1.23E-06 | - | |
| 2-hydroxypalmitate | 31057 | 0.27 ↓ | 3.07E-05 | - | |
| pentadecanoate (15:0) | [00826](http://www.hmdb.ca/metabolites/HMDB00826) | 0.26 ↓ | 0.00066 | - | |
| docosapentaenoate (n3 DPA; 22:5n3) | 01976 | 0.26 ↓ | 0.0048 | - | |
| 5-dodecenoate (12:1n7) | 00529 | 0.26 ↓ | 0.00045 | - | |
| eicosapentaenoate (EPA; 20:5n3) | 01999 | 0.26 ↓ | 0.041 | 0.63 ↓ | 0.010 |
| myristoleate (14:1n5) | 02000 | 0.25 ↓ | 0.0018 | - | |
| 15-methylpalmitate | - | 0.24 ↓ | 0.00014 | - | |
| tetradecanoic acid (myristate, 14:0) | 00806 | 0.24 ↓ | 0.00082 | - | |
| caprate (10:0) | 00511 | 0.24 ↓ | 0.0012 | - | |
| N-palmitoylglycine | - | 0.23 ↓ | 0.00017 | - | |
| suberylglycine | [00953](http://www.hmdb.ca/metabolites/HMDB00953) | 0.23 ↓ | 0.0013 | - | |
| 3-hydroxybutyrate (BHBA) | [00357](http://www.hmdb.ca/metabolites/HMDB00357) | 0.23 ↓ | 0.026 | - | |
| palmitoleate (16:1n7) | 03229 | 0.22 ↓ | 0.00026 | - | |
| dihomo-linoleate (20:2n6) | 05060 | 0.22 ↓ | 0.00028 | - | |
| dihomo-linolenate (20:3n3 or n6) | 02925 | 0.22 ↓ | 0.00047 | - | |
| N-palmitoyltaurine | - | 0.21 ↓ | 0.0020 | - | |
| propionylcarnitine | [00824](http://www.hmdb.ca/metabolites/HMDB00824) | 0.20 ↓ | 0.00015 | - | |
| pristanate | [00795](http://www.hmdb.ca/metabolites/HMDB00795) | 0.20 ↓ | 0.0027 | - | |
| 7-hydroxycholesterol (alpha or beta) | - | 0.20 ↓ | 0.00010 | - | |
| 3b-hydroxy-5-cholenoic acid | [00308](http://www.hmdb.ca/metabolites/HMDB00308) | 0.19 ↓ | 3.69E-05 | - | |
| laurate (12:0) | 00638 | 0.18 ↓ | 8.17E-05 | - | |
| docosadienoate (22:2n6) | 61714 | 0.16 ↓ | 6.88E-06 | - | |
| 3-hydroxymyristate | - | 0.16 ↓ | 7.73E-06 | - | |
| 7-ketolithocholate | [00467](http://www.hmdb.ca/metabolites/HMDB00467) | 0.16 ↓ | 0.0083 | - | |
| erucate (22:1n9) | [02068](http://www.hmdb.ca/metabolites/HMDB02068) | 0.16 ↓ | 4.83E-05 | 0.66 ↓ | 0.037 |
| hyocholate | [00760](http://www.hmdb.ca/metabolites/HMDB00760) | 0.15 ↓ | 0.030 | - | |
| adrenate (22:4n6) | 02226 | 0.15 ↓ | 1.60E-05 | - | |
| 5,8,11-Eicosatrienoic acid (mead acid; 20:3n9) | 10378 | 0.15 ↓ | 0.00017 | 0.57 ↓ | 0.031 |
| beta-muricholate | [00415](http://www.hmdb.ca/metabolites/HMDB00415) | 0.13 ↓ | 0.049 | - | |
| nervonate (24:1n9) | 02368 | 0.12 ↓ | 4.76E-06 | - | |
| cholate sulfate | - | 0.12 ↓ | 5.26E-05 | - | |
| 13-methylmyristate | - | 0.11 ↓ | 0.00010 | - | |
| 5alpha-androstan-3alpha,17beta-diol disulfate | - | 0.11 ↓ | 0.014 | - | |
| N-oleoyltaurine | - | 0.084 ↓ | 0.022 | - | |
| cholate | [00619](http://www.hmdb.ca/metabolites/HMDB00619) | 0.061 ↓ | 0.028 | - | |
| chenodeoxycholate | [00518](http://www.hmdb.ca/metabolites/HMDB00518) | 0.054 ↓ | 0.0078 | - | |
| hyodeoxycholate | [00733](http://www.hmdb.ca/metabolites/HMDB00733) | 0.054 ↓ | 0.0040 | - | |
| caprylate (8:0) | 00482 | - | | 1.33 ↑ | 0.029 |
| suberate (octanedioate) | 00893 | - | | 1.72 ↑ | 0.018 |
| 2-hydroxyglutarate | 00606 | - | | 0.64 ↓ | 0.0087 |
| 3-methyladipate | 00555 | - | | 0.44 ↓ | 0.00086 |
| 2-aminoheptanoate | - | - | | 2.81 ↑ | 0.013 |
| 2-aminooctanoate | 00991 | - | | 0.45 ↓ | 0.016 |
| butyrylcarnitine | [02013](http://www.hmdb.ca/metabolites/HMDB02013) | - | | 0.73 ↓ | 0.041 |
| 3-hydroxybutyrylcarnitine (2) | - | - | | 0.66 ↓ | 0.036 |
| alpha-hydroxycaproate | 01624 | - | | 0.73 ↓ | 0.039 |
| 2-hydroxyoctanoate | 02264 | - | | 0.61 ↓ | 0.039 |
| palmitoyl ethanolamide | [02100](http://www.hmdb.ca/metabolites/HMDB02100) | - | | 0.60 ↓ | 0.035 |
| 1-palmitoyl-2-linoleoyl-glycerophosphoinositol (GPI) (16:0/18:2) | - | - | | 1.54 ↑ | 0.012 |
| 1-stearoyl-2-linoleoyl-glycerophosphoinositol (GPI) (18:0/18:2) | - | - | | 1.33 ↑ | 0.019 |
| 1-palmitoyl-2-stearoyl-glycerophosphocholine (GPC) (16:0/18:0) | - | - | | 1.33 ↑ | 0.017 |
| 1-stearoyl-2-oleoyl-glycerophosphocholine (GPC) (18:0/18:1) | - | - | | 0.73 ↓ | 0.045 |
| 1-palmitoyl-2-palmitoleoyl-glycerophosphocholine (GPC) (16:0/16:1) | - | - | | 0.71 ↓ | 0.028 |
| 1-palmitoyl-2-linolenoyl-glycerophosphocholine (GPC) (16:0/18:3) | - | - | | 0.59 ↓ | 0.015 |
| 1-(1-enyl-stearoyl)-2-linoleoyl-glycerophosphoethanolamine (GPE) (P-18:0/18:2) | - | - | | 1.55 ↑ | 0.021 |
| glycochenodeoxycholate | [00637](http://www.hmdb.ca/metabolites/HMDB00637) | - | | 0.48 ↓ | 0.021 |

* Table displays lipid metabolites with a statistically-significant fold difference between Pro+RB and Pro in both LIC and serum matrices.
** HMDB refers to the Human Metabolome Database, and access numbers are provided for each metabolite identified in the database.
*** For each metabolite, fold difference was calculated by dividing the scaled relative abundance of Pro+RB by Pro, where ↑ indicates that the metabolite had a higher scaled relative abundance in Pro+RB compared to Pro, and ↓ indicates the metabolite had a lower scaled relative abundance in Pro+RB compared to Pro.
