# Supplementary material for: Rice Bran and Probiotics Alter the Porcine Large Intestine and Serum Metabolomes for Protection against Human Rotavirus Diarrhea
Source: Front Microbiol. 2017 Apr 21;8:653. doi: 10.3389/fmicb.2017.00653 (PMC5399067; doi:10.3389/fmicb.2017.00653)
Supplement: Supplementary file 2 [file Table_2.DOCX]

Supplementary Material

Rice bran and Probiotics Alter the Porcine Large Intestinal and Serum Metabolomes for Enhanced Protection against Human Rotavirus Diarrhea

Nora Jean Nealon, Lijuan Yuan, Xingdong Yang and Elizabeth P. Ryan.*

*** Correspondence:** e.p.ryan@colostate.edu

**Supplementary Table 2. Large intestinal content and serum amino acids/peptides in pigs consuming probiotics in the presence and absence of rice bran.**

| **Metabolite^*^** | **HMDB^*^** | **Large Intestinal Contents** | | **Serum** | |
| --- | --- | --- | --- | --- | --- |
|  |  | **Fold Difference^***^** | **p-value** | **Fold Difference** | **p-value** |
| 4-hydroxycinnamate | [02035](http://www.hmdb.ca/metabolites/HMDB02035) | 14.75 ↑ | 1.75E-05 | - | |
| urea | [00294](http://www.hmdb.ca/metabolites/HMDB00294) | 12.83 ↑ | 0.0011 | - | |
| indoleacetate | [00197](http://www.hmdb.ca/metabolites/HMDB00197) | 8.89 ↑ | 0.00028 | - | |
| vanillylmandelate | [00291](http://www.hmdb.ca/metabolites/HMDB00291) | 5.26 ↑ | 2.21E-09 | - | |
| glycylisoleucine | [28844](http://www.hmdb.ca/metabolites/HMDB28844) | 4.08 ↑ | 0.012 | - | |
| 5-methylthioadenosine | 01173 | 3.38 ↑ | 0.0022 | - | |
| gentisate | [00152](http://www.hmdb.ca/metabolites/HMDB00152) | 2.99 ↑ | 0.0025 | - | |
| 4-guanidinobutanoate | [03464](http://www.hmdb.ca/metabolites/HMDB03464) | 2.73 ↑ | 0.0013 | 1.64 ↑ | 0.017 |
| threonylphenylalanine | - | 2.17 ↑ | 0.0039 | - | |
| carnitine | 00062 | 0.68 ↓ | 0.034 | - | |
| imidazole lactate | [02320](http://www.hmdb.ca/metabolites/HMDB02320) | 0.63 ↓ | 0.025 | - | |
| saccharopine | 00279 | 0.58 ↓ | 3.23E-05 | - | |
| S-1-pyrroline-5-carboxylate | [01301](http://www.hmdb.ca/metabolites/HMDB01301) | 0.57 ↓ | 0.026 | - | |
| N-acetylserine | [02931](http://www.hmdb.ca/metabolites/HMDB02931) | 0.57 ↓ | 0.026 | - | |
| guanidinoacetate | [00128](http://www.hmdb.ca/metabolites/HMDB00128) | 0.56 ↓ | 0.011 | 0.64 ↓ | 0.009 |
| pipecolate | 00070 | 0.54 ↓ | 0.016 | - | |
| N6,N6,N6-trimethyllysine | [01325](http://www.hmdb.ca/metabolites/HMDB01325) | 0.49 ↓ | 0.0045 | - | |
| 3-sulfo-L-alanine | [02757](http://www.hmdb.ca/metabolites/HMDB02757) | 0.48 ↓ | 0.011 | - | |
| histidine | [00177](http://www.hmdb.ca/metabolites/HMDB00177) | 0.48 ↓ | 0.012 | - | |
| valine | [00883](http://www.hmdb.ca/metabolites/HMDB00883) | 0.48 ↓ | 0.036 | - | |
| creatine | [00064](http://www.hmdb.ca/metabolites/HMDB00064) | 0.47 ↓ | 0.0078 | - | |
| gamma-aminobutyrate (GABA) | [00112](http://www.hmdb.ca/metabolites/HMDB00112) | 0.46 ↓ | 0.023 | - | |
| cysteine sulfinic acid | [00996](http://www.hmdb.ca/metabolites/HMDB00996) | 0.45 ↓ | 0.015 | - | |
| dimethylarginine (Symmetrical and Asymmetrical) | [01539](http://www.hmdb.ca/metabolites/HMDB01539) | 0.44 ↓ | 0.0012 | - | |
| homoarginine | [00670](http://www.hmdb.ca/metabolites/HMDB00670) | 0.43 ↓ | 0.049 | - | |
| methionine sulfoxide | [02005](http://www.hmdb.ca/metabolites/HMDB02005) | 0.42 ↓ | 0.0085 | - | |
| argininosuccinate | [00052](http://www.hmdb.ca/metabolites/HMDB00052) | 0.42 ↓ | 0.0017 | - | |
| 4-hydroxyglutamate | [01344](http://www.hmdb.ca/metabolites/HMDB01344) | 0.42 ↓ | 0.049 | - | |
| cystathionine | [00099](http://www.hmdb.ca/metabolites/HMDB00099) | 0.41 ↓ | 0.0019 | - | |
| homocitrulline | [00679](http://www.hmdb.ca/metabolites/HMDB00679) | 0.41 ↓ | 0.016 | - | |
| homocysteine | [00742](http://www.hmdb.ca/metabolites/HMDB00742) | 0.41 ↓ | 0.029 | - | |
| kynurenate | [00715](http://www.hmdb.ca/metabolites/HMDB00715) | 0.40 ↓ | 0.0015 | - | |
| cysteine | [00574](http://www.hmdb.ca/metabolites/HMDB00574) | 0.40 ↓ | 0.033 | 0.62 ↓ | 0.014 |
| alanine | [00161](http://www.hmdb.ca/metabolites/HMDB00161) | 0.40 ↓ | 0.026 | - | |
| 1-methylhistamine | [00898](http://www.hmdb.ca/metabolites/HMDB00898) | 0.40 ↓ | 0.044 | 1.40 ↑ | 0.010 |
| aspartate | [00191](http://www.hmdb.ca/metabolites/HMDB00191) | 0.38 ↓ | 0.0029 | - | |
| trans-urocanate | [00301](http://www.hmdb.ca/metabolites/HMDB00301) | 0.38 ↓ | 0.0022 | - | |
| 2-aminobutyrate | [00650](http://www.hmdb.ca/metabolites/HMDB00650) | 0.38 ↓ | 0.031 | - | |
| N-acetylcarnosine | [12881](http://www.hmdb.ca/metabolites/HMDB12881) | 0.37 ↓ | 3.92E-06 | - | |
| ornithine | [03374](http://www.hmdb.ca/metabolites/HMDB03374) | 0.37 ↓ | 0.040 | 0.71 ↓ | 0.047 |
| glutamate | [00148](http://www.hmdb.ca/metabolites/HMDB00148) | 0.36 ↓ | 2.38E-05 | - | |
| N-acetylputrescine | [02064](http://www.hmdb.ca/metabolites/HMDB02064) | 0.36 ↓ | 0.00028 | - | |
| anserine | [00194](http://www.hmdb.ca/metabolites/HMDB00194) | 0.36 ↓ | 2.04E-06 | - | |
| N-acetylhistidine | [32055](http://www.hmdb.ca/metabolites/HMDB32055) | 0.36 ↓ | 0.046 | 1.64 ↑ | 0.0025 |
| N-acetylthreonine | - | 0.35 ↓ | 0.017 | - | |
| citrulline | [00904](http://www.hmdb.ca/metabolites/HMDB00904) | 0.35 ↓ | 0.047 | 0.83 ↓ | 0.048 |
| 5-oxoproline | [00267](http://www.hmdb.ca/metabolites/HMDB00267) | 0.34 ↓ | 0.0018 | - | |
| N-acetylhistamine | [13253](http://www.hmdb.ca/metabolites/HMDB13253) | 0.34 ↓ | 0.014 | 1.66 ↑ | 0.024 |
| N6-carboxyethyllysine | - | 0.33 ↓ | 0.0010 | - | |
| ophthalmate | [05765](http://www.hmdb.ca/metabolites/HMDB05765) | 0.33 ↓ | 0.0026 | - | |
| proline | [00162](http://www.hmdb.ca/metabolites/HMDB00162) | 0.31 ↓ | 0.0069 | - | |
| gamma-glutamylleucine | [11171](http://www.hmdb.ca/metabolites/HMDB11171) | 0.31 ↓ | 0.025 | - | |
| 5-hydroxylysine | [00450](http://www.hmdb.ca/metabolites/HMDB00450) | 0.30 ↓ | 0.0017 | 0.76 ↓ | 0.011 |
| putrescine | 01414 | 0.30 ↓ | 9.20E-08 | - | |
| N-acetylalanine | [00766](http://www.hmdb.ca/metabolites/HMDB00766) | 0.30 ↓ | 0.023 | - | |
| cadaverine | [02322](http://www.hmdb.ca/metabolites/HMDB02322) | 0.29 ↓ | 8.58E-06 | - | |
| N-acetylasparagine | [06028](http://www.hmdb.ca/metabolites/HMDB06028) | 0.28 ↓ | 0.0040 | - | |
| 3-hydroxyanthranilate | [01476](http://www.hmdb.ca/metabolites/HMDB01476) | 0.28 ↓ | 0.016 | - | |
| 3-methylhistidine | [00479](http://www.hmdb.ca/metabolites/HMDB00479) | 0.26 ↓ | 2.70E-05 | - | |
| carnosine | [00033](http://www.hmdb.ca/metabolites/HMDB00033) | 0.26 ↓ | 0.00043 | - | |
| N-acetyltaurine | - | 0.26 ↓ | 0.00051 | - | |
| guanidinosuccinate | [03157](http://www.hmdb.ca/metabolites/HMDB03157) | 0.25 ↓ | 0.00027 | - | |
| N-acetyl-cadaverine | - | 0.24 ↓ | 9.36E-05 | - | |
| N1,N12-diacetylspermine | [02172](http://www.hmdb.ca/metabolites/HMDB02172) | 0.24 ↓ | 0.00012 | - | |
| 1-methylguanidine | [01522](http://www.hmdb.ca/metabolites/HMDB01522) | 0.24 ↓ | 0.00084 | 0.53 ↓ | 0.034 |
| lysine | 00182 | 0.23 ↓ | 0.00071 | - | |
| taurine | [00251](http://www.hmdb.ca/metabolites/HMDB00251) | 0.21 ↓ | 0.00034 | - | |
| gamma-glutamylhistidine | - | 0.21 ↓ | 0.0023 | - | |
| glycine | [00123](http://www.hmdb.ca/metabolites/HMDB00123) | 0.20 ↓ | 0.0021 | - | |
| N-acetylglutamate | [01138](http://www.hmdb.ca/metabolites/HMDB01138) | 0.20 ↓ | 0.0030 | - | |
| N-methylhydantoin | [03646](http://www.hmdb.ca/metabolites/HMDB03646) | 0.20 ↓ | 0.0039 | - | |
| C-glycosyltryptophan | - | 0.19 ↓ | 3.52E-06 | - | |
| histamine | [00870](http://www.hmdb.ca/metabolites/HMDB00870) | 0.18 ↓ | 0.00030 | 1.57 ↑ | 0.043 |
| N-acetylmethionine sulfoxide | - | 0.18 ↓ | 0.038 | - | |
| proline-hydroxy-proline | [06695](http://www.hmdb.ca/metabolites/HMDB06695) | 0.18 ↓ | 0.032 | 1.38 ↑ | 0.00014 |
| N-formylmethionine | [01015](http://www.hmdb.ca/metabolites/HMDB01015) | 0.17 ↓ | 0.010 | - | |
| formiminoglutamate | - | 0.16 ↓ | 0.00061 | - | |
| N-acetylcitrulline | [00856](http://www.hmdb.ca/metabolites/HMDB00856) | 0.16 ↓ | 0.0027 | - | |
| N-acetylmethionine | [11745](http://www.hmdb.ca/metabolites/HMDB11745) | 0.16 ↓ | 0.027 | - | |
| N6-acetyllysine | 00206 | 0.15 ↓ | 0.00014 | - | |
| gamma-glutamylvaline | [11172](http://www.hmdb.ca/metabolites/HMDB11172) | 0.15 ↓ | 0.0011 | - | |
| N-acetylleucine | [11756](http://www.hmdb.ca/metabolites/HMDB11756) | 0.15 ↓ | 0.036 | - | |
| gamma-glutamylisoleucine | [11170](http://www.hmdb.ca/metabolites/HMDB11170) | 0.14 ↓ | 0.00086 | - | |
| N-acetylkynurenine | - | 0.14 ↓ | 2.57E-05 | 2.01 ↑ | 0.021 |
| homogentisate | [00130](http://www.hmdb.ca/metabolites/HMDB00130) | 0.14 ↓ | 0.0011 | - | |
| N-formylphenylalanine | - | 0.14 ↓ | 0.016 | - | |
| N-acetylvaline | [11757](http://www.hmdb.ca/metabolites/HMDB11757) | 0.13 ↓ | 0.0010 | - | |
| gamma-glutamyltyrosine | [11741](http://www.hmdb.ca/metabolites/HMDB11741) | 0.13 ↓ | 0.0028 | - | |
| N-acetylaspartate | [00812](http://www.hmdb.ca/metabolites/HMDB00812) | 0.11 ↓ | 7.12E-05 | 0.76 ↓ | 0.025 |
| N-acetylphenylalanine | [00512](http://www.hmdb.ca/metabolites/HMDB00512) | 0.075 ↓ | 0.0031 | - | |
| pyroglutamine | - | 0.074 ↓ | 0.0077 | 0.43 ↓ | 0.021 |
| N2-acetyllysine | 00446 | 0.07 ↓ | 0.0068 | - | |
| gamma-glutamylglutamate | [11737](http://www.hmdb.ca/metabolites/HMDB11737) | 0.063 ↓ | 1.62E-05 | - | |
| gamma-glutamylalanine | [29142](http://www.hmdb.ca/metabolites/HMDB29142) | 0.063 ↓ | 8.15E-05 | - | |
| gamma-glutamyl-epsilon-lysine | [03869](http://www.hmdb.ca/metabolites/HMDB03869) | 0.061 ↓ | 2.18E-05 | - | |
| gamma-glutamylglycine | [11667](http://www.hmdb.ca/metabolites/HMDB11667) | 0.058 ↓ | 3.85E-05 | - | |
| imidazole propionate | [02271](http://www.hmdb.ca/metabolites/HMDB02271) | 0.058 ↓ | 0.038 | - | |
| prolylglycine | - | 0.057 ↓ | 0.0015 | 0.44 ↓ | 0.014 |
| gamma-glutamylmethionine | [29155](http://www.hmdb.ca/metabolites/HMDB29155) | 0.045 ↓ | 0.00019 | - | |
| spermidine | [01257](http://www.hmdb.ca/metabolites/HMDB01257) | 0.038 ↓ | 1.64E-10 | - | |
| N(1)-acetylspermine | [01186](http://www.hmdb.ca/metabolites/HMDB01186) | 0.038 ↓ | 1.03E-09 | - | |
| N-acetyltyrosine | [00866](http://www.hmdb.ca/metabolites/HMDB00866) | 0.034 ↓ | 0.00028 | - | |
| phenyllactate (PLA) | [00779](http://www.hmdb.ca/metabolites/HMDB00779) | - | | 2.08 ↑ | 0.0031 |
| 1-methylimidazoleacetate | [02820](http://www.hmdb.ca/metabolites/HMDB02820) | - | | 1.57 ↑ | 0.0020 |
| S-methylcysteine | [02108](http://www.hmdb.ca/metabolites/HMDB02108) | - | | 1.48 ↑ | 0.013 |
| 4-acetamidobutanoate | [03681](http://www.hmdb.ca/metabolites/HMDB03681) | - | | 1.34 ↑ | 0.018 |
| dimethylglycine | 00092 | - | | 1.34 ↑ | 0.038 |
| cystine | [00192](http://www.hmdb.ca/metabolites/HMDB00192) | - | | 1.30 ↑ | 0.044 |
| trans-4-hydroxyproline | [00725](http://www.hmdb.ca/metabolites/HMDB00725) | - | | 1.14 ↑ | 0.046 |
| creatinine | [00562](http://www.hmdb.ca/metabolites/HMDB00562) | - | | 0.89 ↓ | 0.035 |
| thyroxine | [01918](http://www.hmdb.ca/metabolites/HMDB01918) | - | | 0.73 ↓ | 0.023 |
| glutamine | [00641](http://www.hmdb.ca/metabolites/HMDB00641) | - | | 0.70 ↓ | 0.017 |
| N-acetylglutamine | [06029](http://www.hmdb.ca/metabolites/HMDB06029) | - | | 0.71 ↓ | 0.031 |
| hypotaurine | [00965](http://www.hmdb.ca/metabolites/HMDB00965) | - | | 0.65 ↓ | 0.028 |
| N-delta-acetylornithine | - | - | | 0.52 ↓ | 0.014 |

* Table displays amino acid/peptide metabolites with a statistically-significant fold difference between Pro+RB and Pro in both LIC and serum matrices.
** HMDB refers to the Human Metabolome Database, and access numbers are provided for each metabolite identified in the database.
***For each metabolite, fold difference was calculated by dividing the scaled relative abundance of Pro+RB by Pro, where ↑ indicates that the metabolite had a higher scaled relative abundance in Pro+RB compared to Pro, and ↓ indicates the metabolite had a lower scaled relative abundance in Pro+RB compared to Pro.
