# Supplementary material for: Rice Bran and Probiotics Alter the Porcine Large Intestine and Serum Metabolomes for Protection against Human Rotavirus Diarrhea
Source: Front Microbiol. 2017 Apr 21;8:653. doi: 10.3389/fmicb.2017.00653 (PMC5399067; doi:10.3389/fmicb.2017.00653)
Supplement: Supplementary file 3 [file Table_3.DOCX]

Supplementary Material

Rice bran and Probiotics Alter the Porcine Large Intestinal and Serum Metabolomes for Enhanced Protection against Human Rotavirus Diarrhea

Nora Jean Nealon, Lijuan Yuan, Xingdong Yang, and Elizabeth P. Ryan*

*** Correspondence:** e.p.ryan@colostate.edu
 **Supplementary Table 3. Large intestinal content and serum lipids with immunomodulatory, gut barrier protective and antiviral functions related to anti-diarrheal activity.**

|  | **Large Intestinal Contents** | | **Serum** | | **Functions** | **References** |
| --- | --- | --- | --- | --- | --- | --- |
| **Lipid*** | **Fold Difference**** | **p-value** | **Fold Difference** | **p-value** |  |  |
| 1-linoleoylglycerol (18:2) | 320.72 ↑ | 1.04E-08 | - | - | Has been demonstrated to systemically reduce inflammation in porcine models. | Han *et al*., 2015 |
| 2-oleoylglycerol | 293.40 ↑ | 3.04E-10 | - | - | Influences gastric emptying via modulation of cholecystekinin release. | MandØe *et al.*, 2015 |
| 2-linoleoyl glycerol (18:2) | 187.09 ↑ | 9.27E-10 | - | - | Serves as an important precursor of 2-arachidonyl glycerol that goes on to modulate inflammation through direct binding to endocannabinoid receptors, or via its catabolism into ligands of cyclooxygenases, cytochrome P450 enzymes, and lipoxygenase enzymes. | [Mechoulam *et al.*, 1995](#_ENREF_15), [Turcotte *et al*., 2015](#_ENREF_29) |
| beta-sitosterol | 15.59 ↑ | 4.72E-11 | - | - | As part of an herbal extract, it acted on gut muscarinic and histamatergic receptors to reduce gut motility associated with diarrhea. | [Mehmood *et al.*, 2014](#_ENREF_16) |
| 2-palmitoylglycerol | 7.08 ↑ | 6.33E-05 | - | - | Produced by mammalian and bacterial cells and influences cannabinoid-1 receptor expression in peripheral organs, including the gut, and thereby influences intestinal motility. | [Yuan *et al.*, 2016](#_ENREF_31), [Murataeva *et al.*, 2016](#_ENREF_17), [Aviello *et al.*, 2008](#_ENREF_3) |
| palmitoyl sphingomyelin (d18:1/16:0) | 5.42 ↑ | 0.017 | - | - | Influences the composition of the intestinal microbiome and colon mucosal cell shedding. | [Sinha *et al.*, 2016](#_ENREF_25) |
| 12,13-dihydroxyoctadecenoic acid (DiHOME) | 5.26 ↑ | 0.0012 | - | - | Influences the composition of the neonatal intestinal microbiome and modulates T-regulatory cell development. | [Fujimura *et al.*, 2016](#_ENREF_9) |
| heptanedioate (pimelate) | 5.17 ↑ | 0.00033 | 1.58 ↑ | 0.010 | Acts to increase butyrate and biotin metabolism in the gut lumen, and thus indirectly influences colonic T-regulatory cell populations. | [Sugahara *et al.*, 2015](#_ENREF_27) |
| malonate | 4.81 ↑ | 1.26E-06 | - | - | As part of a fruit extract, exhibited anti-diarrheal properties in humans. | [Pandey *et al.*, 2016](#_ENREF_20) |
| linoleoyl ethanolamide | 4.42 ↑ | 0.0014 | - | - | Present in plants and has demonstrated anti-inflammatory effects in vitro and in vivo by suppressing interleukin 1B, 6, tumor necrosis factor alpha, cyclooxygenase enzyme-2, and toll-like receptor 4, and nuclear factor kB expression in macrophages. | [Ishida *et al.*, 2013](#_ENREF_11) |
| nonanedioate (azelate) | 4.23 ↑ | 0.0015 | 1.73 ↑ | 0.023 | Associated with decreased pro-inflammatory interleukin-6 expression in the serum of human adults. | [Lustgarten and Fielding, 2016](#_ENREF_14) |
| 13 + 9 hydroxyoctadecadienoic acid (13 + 9 HODE) | 3.93 ↑ | 0.010 | - | - | In humans, 13 and 9-HODE are released by peripheral blood populations of neutrophils, eosinophils, basophils, monocytes and lymphocytes to modulate inflammatory and immune processes. 9-HODE has demonstrated anti-inflammatory properties by scavenging free radicals. | [Engels *et al.*, 1996](#_ENREF_7), [Collino *et al.*, 2013](#_ENREF_5) |
| choline phosphate | 3.43 ↑ | 0.0027 | 1.61 ↑ | 0.046 | Provides structural integrity to cell membranes, including the mucosal lining of the intestinal tract. | [Liu *et al.*, 2014](#_ENREF_13) |
| mevalonate | 2.44 ↑ | 0.013 | - | - | Modulates innate immune responses by influencing interleukin 1-B production | [Akula *et al.*, 2016](#_ENREF_1) |
| stearate (18:0) | 0.65 ↓ | 0.015 | - | - | As part of a plant oil supplement, it reduced castor-oil induced murine diarrhea. | [Zavala-Mendoza *et* *al.*, 2013](#_ENREF_32) |
| palmitate (16:0) | 0.59 ↓ | 0.0087 | - | - | As part of a plant oil supplement, it reduced castor-oil induced murine diarrhea. | [Zavala-Mendoza *et al.*, 2013](#_ENREF_32) |
| oleate (vaccenate) (18:1) | 0.56 ↓ | 0.030 | - | - | As part of plant oil supplements, it reduced diarrhea and inflammation in an experimentally-induced rat colitis model and also in a castor-oil induced murine diarrhea model. In humans, it slowed gastrointestinal time and reduced diarrhea by activating nutrient-triggered inhibitory feedback mechanisms in the small intestine responsible for gut motility. | [Lin *et al.*, 2001](#_ENREF_12), [Zavala-Mendoza *et al*., 2013](#_ENREF_32), [Naouar *et* *al.*, 2016](#_ENREF_18) |
| sphingosine | 0.53 ↓ | 0.029 | - | - | Converted in the gut into the bioactive intermediate sphingosine-1-phosphate that modulates mucosal lymphocyte trafficking, influences mucosal cell proliferation, promotes gut integrity, and increases populations of IgA-producing intraepithelial lymphocytes. Also converted within intestinal epithelial cells to palmitate, which may reduce diarrhea arising from inflammatory processes. | [Zavala-Mendoza *et al.*, 2013](#_ENREF_32), [Nilsson, 2016](#_ENREF_19) |
| cholesterol | 0.36 ↓ | 0.00052 | - | - | In a rat induced-colitis model of diarrhea being treated with *Lactobacillus plantarum*, protection against diarrhea was associated with lower colonic lumen levels of cholesterol, and its presence in the intestinal lumen was inversely related to diarrhea in children with rotavirus-induced diarrhea. | [Alp Avci, 2016](#_ENREF_2), [Trabelsi *et al.*, 2016](#_ENREF_28) |
| docosapentanaenoate (n3 DPA; 22:5n3) | 0.26 ↓ | 0.0048 | - | - | Lower intestinal luminal levels have been correlated with decreased inflammation of the gut mucosa, and this metabolite has been demonstrated to modulate ileal contractility. | [Patten *et al.*, 2002](#_ENREF_21), [Pearl *et al.*, 2014](#_ENREF_22) |
| eicosapentaenoate (EPA; 20:5n3) | 0.26 ↓ | 0.041 | - | - | When fed as an oral supplement to mice, it enhanced helper T-cell function. | [Wee *et al.*, 1988](#_ENREF_30) |
| caprate (10:0) | 0.24 ↓ | 0.00082 | - | - | Concentration in human intestinal lumen is inversely associated with tight junction integrity. | [Soderholm *et al.*, 2002](#_ENREF_26) |
| palmitoleate (16:1n7) | 0.22 ↓ | 0.00026 | - | - | In humans, oral intake was associated with decreased gastric emptying times, and evidence supports microbial generation of this metabolite. In mice given dietary supplementation, it reduced the expression of pro-inflammatory cytokines tumor necrosis factor alpha, interleukin-1B, 6, and 8. | [Schirmer *et al.*, 2016](#_ENREF_24), [Frigolet and Gutiérrez-Aguilar, 2017](#_ENREF_8) |
| chenodeoxycholate | 0.054 ↓ | 0.0078 | - | - | Lower levels in the colonic lumen may be associated with decreased motility and longer digests transit time. | [Goyal *et al.*, 2015](#_ENREF_10), [Peleman *et al.*, 2016](#_ENREF_23) |
| hyodeoxycholate | 0.054 ↓ | 0.0040 | - | - | Rodent studies suggest that it may act directly on colonic mucosal goblet cells to increase mucus production, thereby modulating barrier function. | [Barcelo *et al.*, 2001](#_ENREF_4) |
| palmitoyl ethanolamide | - | - | 0.60 ↓ | 0.035 | In the gastrointestinal tract, reduces inflammation by inhibiting mast cell degranulation, and modulates the enteric nervous system via inhibiting the degradation of the endocannabinoid anandamide to influence gut motility. | [Cordaro *et al.*, 2016](#_ENREF_6) |

* Table displays lipid metabolites with a statistically-significant fold difference between Pro+RB and Pro in both LIC and Serum matrices that were determined to have anti-diarrheal properties after a comprehensive peer-reviewed literature search.
** For each metabolite, fold difference was calculated by dividing the scaled relative abundance of Pro+RB by Pro, where ↑ indicates that the metabolite had a higher scaled relative abundance in Pro+RB compared to Pro, and ↓ indicates the metabolite had a lower scaled relative abundance in Pro+RB compared to Pro.
