# Supplementary material for: Rice Bran and Probiotics Alter the Porcine Large Intestine and Serum Metabolomes for Protection against Human Rotavirus Diarrhea
Source: Front Microbiol. 2017 Apr 21;8:653. doi: 10.3389/fmicb.2017.00653 (PMC5399067; doi:10.3389/fmicb.2017.00653)
Supplement: Supplementary file 4 [file Table_4.docx]

Supplementary Material

Rice bran and Probiotics Alter the Porcine Large Intestinal and Serum Metabolomes for Enhanced Protection against Human Rotavirus Diarrhea

Nora Jean Nealon, Lijuan Yuan, Xingdong Yang, and Elizabeth P. Ryan*

*** Correspondence:** e.p.ryan@colostate.edu

**Supplementary Table 4. Large intestinal content and serum amino acids/peptides with immunomodulatory, gut barrier protective and antiviral functions related to anti-diarrheal activity.**

|  | **Large Intestinal Contents** | | **Serum** | | **Functions** | **References** |
| --- | --- | --- | --- | --- | --- | --- |
| **Amino Acid/Peptide*** | **Fold Difference**** | **p-value** | **Fold Difference** | **p-value** |  |  |
| histidine | 0.49 ↓ | 0.012 | - | - | As part of a rice-based oral rehydration solution, functioned as an anti-secretory agent to reduce *C*holera-associated diarrhea in adult males. | Rabanni *et al*., 2005 |
| gamma-aminobutyrate (GABA) | 0.46 ↓ | 0.024 | - | - | In the mammalian enteric nervous system, GABA-ergic signaling is important in modulating intestinal fluid balance, gut motility and inflammation. | Li *et al.*, 2012; Auteri *et al.*, 2015 |
| cysteine | 0.41 ↓ | 0.033 | 0.63 ↓ | 0.014 | Reduced oxidative stress, decreased inflammation, lowered permeability and modulated the mucosal immune system in the intestinal tract of neonatal pigs. | Bauchart-Thevret *et al.*, 2009; Ruth and Field, 2013 |
| homocysteine | 0.41 ↓ | 0.029 | - | - | Potentiated the activity of S-adenosyl hydrolase antiviral drugs against vaccinia and vesicular stomatitis viruses. | Hasobe *et al.*, 1989 |
| alanine | 0.40 ↓ | 0.026 | - | - | When supplemented into an oral rehydration salt in adult males, reduced diarrhea associated with Cholera and ETEC *E. coli*. | Patra *et al.*, 1989 |
| glutamate | 0.37 ↓ | 2.39 E-05 | - | - | Supplementation prevented diarrhea in rats challenged with a diarrhea-inducing intra-gastric tube diet, and reduced diarrhea in neonatal pigs at 1-week post-weaning. | [Somekawa *et al.*, 2012](#_ENREF_16), [Rezaei *et al.*, 2013](#_ENREF_14) |
| ornithine | 0.37 ↓ | 0.041 | 0.71 ↓ | 0.047 | In calves supplemented with lysine, increased fecal shedding of ornithine was associated with increased diarrhea, and modulations to ornithine metabolism are associated with decreased in-vitro and in-vivo replication of multiple RNA viruses. | [Abe *et al.*, 2001](#_ENREF_1), Mounce *et al.*, 2016 |
| citrulline | 0.36 ↓ | 0.047 | 0.84 ↓ | 0.048 | Serum levels are an indicator of functional enterocytes mass and citrulline has been used as a biomarker for assessing mucosal barrier dysfunction. | [Kong *et al.*, 2015](#_ENREF_8), [Wang *et al.*, 2015](#_ENREF_20) |
| proline | 0.31 ↓ | 0.0069 | - | - | Prophylactic dietary supplementation in mice infected with porcine circovirus-2 was associated with enhanced innate and adaptive immune responses, reduced microscopic lesion scores, and lower viral loads. | [Ren *et al.*, 2013](#_ENREF_13) |
| 3-methylhistidine | 0.27 ↓ | 2.70E-05 | - | - | Used as a marker of tissue protein breakdown, where lower levels were indirectly indicative of improved tissue integrity. | Nielsch *et al.*, 1991 |
| carnosine | 0.27 ↓ | 0.0004 | - | - | In humans, decreased diarrhea due to irritable bowel syndrome. | Baraniuk *et al*., 2013 |
| taurine | 0.21 ↓ | 0.0003 | - | - | In mice experimentally induced with colitis, supplementation attenuated the severity of the diarrhea, improved histopathology scores, and inhibited TNF-α and IL-8 production. | Shimizu *et al*., 2009 |
| histamine | 0.19 ↓ | 0.0003 | - | - | A key modulator of intestinal mucosal immune homeostasis in states of health and inflammation. | Smolinska *et al*., 2014 |
| gamma-glutamylvaline | 0.16 ↓ | 0.0011 | - | - | Functions as a ligand for the intestinal extracellular calcium sensing receptor, where binding leads to reductions in TNF- α. IL-8, IL-6, IL-17 and IL-1B and increases in IL-10 in the colon. | [Zhang *et al.*, 2015](#_ENREF_22) |
| gamma-glutamyl-epsilon-lysine | 0.06 ↓ | 2.19E-05 | - | - | Presence is associated with the repair of the colonic mucosa during inflammatory ulcerative processes. | [D'Argenio *et al.*, 2005](#_ENREF_4) |
| spermidine | 0.04 ↓ | 1.03E-09 | - | - | Influences the development of the intestinal tract postnatally, influences enterocyte protein and nuclei acid metabolism, and may modulate mucosal immunity. | [Plaza-Zamora *et al.*, 2013](#_ENREF_12), [Timmons *et al.*, 2012](#_ENREF_19) |
| cystine | - | - | 1.30 ↑ | 0.044 | In humans and rodents, serves as an excitatory neurotransmitter in the brainstem where it functions downstream to delay gastric emptying and suppresses food intake and also functions as a gut extracellular antioxidant that can improve the viability of probiotic species. | [Khan *et al.*, 2014](#_ENREF_7), [McGavigan *et al.*, 2015](#_ENREF_10) |
| trans-4-hydroxyproline | - | - | 1.15 ↑ | 0.046 | In neonatal pigs, may be a multi-systemic indicator of tissue collagen remodeling and growth. | [Brundige *et al.*, 2010](#_ENREF_3) |
| thyroxine | - | - | 0.74 ↓ | 0.023 | Influences gastric acid secretion and modulate nutrient absorption across the small intestine, including decreasing glucose uptake into enterocytes. Modulates the sucrose-isomaltase brush border activity in the small intestine, which may interfere with rotavirus pathogenicity. | [Matty and Seshadri, 1965](#_ENREF_9), [Yeh *et al.*, 1989](#_ENREF_21), [Jourdan *et al.*, 1998](#_ENREF_6) |
| butyrylcarnitine | - | - | 0.73 ↓ | 0.041 | *In vitro* studies support that it may reduce inflammation by promoting normal fatty acid oxidation, deregulation of which is associated with colonic inflammation. | [Srinivas *et al.*, 2007](#_ENREF_17) |
| glutamine | - | - | 0.71 ↓ | 0.017 | When provided as a dietary supplement to rats, reduced secretory diarrhea due to cholera toxin, and in humans and animals, supplementation reduces intestinal damage and improves mucosal immunity. | [Gutiérrez *et al.*, 2007](#_ENREF_5), [Swami *et al.*, 2013](#_ENREF_18) |
| hypotaurine | - | - | 0.66 ↓ | 0.028 | Functions as an osmoprotectant to reduce hyperosmotic stress and the associated inflammation. | [Brocker *et al.,* 2012](#_ENREF_2) |

* Table displays amino acid and peptide metabolites with a statistically-significant fold difference between Pro+RB and Pro in both LIC and Serum matrices that were determined to have anti-diarrheal properties after a comprehensive peer-reviewed literature search.
** For each metabolite, fold difference was calculated by dividing the scaled relative abundance of Pro+RB by Pro, where ↑ indicates that the metabolite had a higher scaled relative abundance in Pro+RB compared to Pro, and ↓ indicates the metabolite had a lower scaled relative abundance in Pro+RB compared to Pro.
